# Temperature-dependent molecular sieving of fluorinated propane/propylene mixtures by a flexible-robust metal-organic framework

**DOI:** 10.1126/sciadv.adj6473

**Published:** 2024-01-19

**Authors:** Wei Xia, Yisi Yang, Liangzheng Sheng, Zhijie Zhou, Lihang Chen, Zhangjing Zhang, Zhiguo Zhang, Qiwei Yang, Qilong Ren, Zongbi Bao

**Affiliations:** ^1^ Key Laboratory of Biomass Chemical Engineering of Ministry of Education, College of Chemical and Biological Engineering, Zhejiang University, 310027 Hangzhou, P. R. China.; ^2^ Institute of Zhejiang University-Quzhou, 324000 Quzhou, P. R. China.; ^3^ Fujian Provincial Key Laboratory of Polymer Materials, College of Materials Science and Engineering, Fujian Normal University, 350007 Fuzhou, P. R. China.

## Abstract

The electronics industry necessitates highly selective adsorption separation of hexafluoropropylene (C_3_F_6_) from perfluoropropane (C_3_F_8_), which poses a challenge due to their similar physiochemical properties. In this work, we present a microporous flexible-robust metal-organic framework (Ca-tcpb) with thermoregulatory gate opening, a rare phenomenon that allows tunable sieving of C_3_F_8_/C_3_F_6_. Remarkably, the temperature-dependent adsorption behavior enhances the discrimination between the larger C_3_F_8_ and the smaller C_3_F_6_, resulting in unprecedented C_3_F_6_/C_3_F_8_ selectivity (over 10,000) compared to other well-known porous materials at an optimal temperature (298 K). Dynamic breakthrough experiments demonstrate that high-purity C_3_F_8_ (over 99.999%) could be obtained from a C_3_F_6_/C_3_F_8_ (10:90) mixture under ambient conditions. The unique attributes of this material encompass exceptional adsorption selectivity, remarkable structural stability, and outstanding separation performance, positioning it as a highly promising candidate for C_3_F_6_/C_3_F_8_ separation. Single-crystal structural analysis of C_3_F_6_-loaded Ca-tcpb and theoretical calculations elucidate the host-guest interaction via multiple intermolecular interactions.

## Teaser

Temperature-dependent sieving of C_3_F_8_ from C_3_F_6_ was realized by a flexible-robust metal-organic framework.

## INTRODUCTION

Electronic specialty gases, such as fluorinated gases (F-gases), are essential for the semiconductor intelligent manufacturing industry, such as the production of ultralarge-scale integrated circuits, solar cells, and other devices. The global market for these gases is expected to exceed $6 billion by 2025 (*1*, *2*). Sulfur hexafluoride, nitrogen trifluoride, and fluorohydrocarbons (e.g., CF_4_, C_2_F_6_, and C_3_F_8_) are among the most widely used F-gases, accounting for about one-third of the overall consumption (*3*). Perfluoropropane (C_3_F_8_) is particularly in high demand as a plasma etching and cleaning gas for semiconductor fabrication (*4*). However, the conventional production of C_3_F_8_ by adding fluorine to hexafluoropropylene (C_3_F_6_) results in a mixture containing 1 to 10% of residual C_3_F_6_, which requires further purification to achieve the desired high purity (more than 99.999%) of C_3_F_8_ (*5*, *6*). The traditional distillation method is energy-intensive and inefficient due to the small difference in the volatilities of C_3_F_6_ and C_3_F_8_ (boiling points: C_3_F_6_, 236.3 K; C_3_F_8_, 242 K). Therefore, there is an urgent need for energy-efficient technologies capable of separating C_3_F_6_ from C_3_F_8_ at ambient conditions, such as physisorption using porous materials (*7*).

The adsorptive separation of C_3_F_6_ from C_3_F_8_ is one of the most difficult challenges in the field of adsorbents, similar to the case of olefin/paraffin separation (*8*). Compared to conventional carbon materials (*9*, *10*) and zeolites (*11**–**13*), metal-organic framework materials (MOFs) or porous coordination polymers (PCPs) offer more diversity and modularity in their organic moieties, making them promising candidates for this task (*14**–**19*). Two well-established strategies have been proposed to enhance the halocarbon capture and separation performance of MOFs (*20*): (i) introducing open metal sites (OMSs) (*21**–**26*) that can form π-complexation with the adsorbates and (ii) functionalizing the ligands with fluorine atoms (*27**–**30*) that can create electron-deficient aromatic rings and increase the host-guest interaction. However, these strategies tend to focus on improving the adsorption capacity of MOFs, which may also increase the uptake of impurities and reduce the selectivity.

The ideal adsorptive separation is based on the size exclusion effect, which can achieve the highest selectivity and efficiency compared to thermodynamic/kinetic separation (*31*, *32*). Several adsorbents have been applied for the size exclusion separation of alkane/alkene/alkyne (*33**–**43*), alkane isomers (*44**–**46*), styrene/ethylbenzene (*47*), and xylene isomers (*48*). However, no such adsorbents have been reported for C_3_F_6_/C_3_F_8_ or any other F-gases so far. The simple analogy to common olefins such as C3 (propylene/propane) is not applicable, because the fluorine atom introduces unique electronegativity, electronic effects, and steric hindrance that make the differences between C_3_F_6_ and C_3_F_8_ very subtle (C_3_F_6_, 7.3 Å × 6.2 Å × 5.1 Å; C_3_F_8_, 7.6 Å × 5.3 Å × 5.1; fig. S1A) (*49*). Size exclusion is rather tricky because it requires precise control (e.g., pore sizes and shapes) of the adsorbents (*50*). Moreover, the adsorption-desorption kinetics and uptake are usually slow and low in confined spaces, which limits the use of rigid frames for pore editing at sub-angstrom level (*33**–**35*, *42*). To address this problem, some researchers have recently fulfilled flexible PCPs with gating effects that can achieve molecular sieving for the intricate separation systems (*51**–**53*) [e.g., ethylene/ethane (*54*) and propylene/propane (*55*)]. For instance, the MOF JNU-3a exhibits dynamic molecular sieving with orthogonal-array pores that open differently to propylene and propane at substantially different temperatures. This allows not only high selectivity but also fast diffusion. Furthermore, we can tune the gate-opening pressure by temperature and facilitate complete separation of binary (*56*, *57*) or even multi-component mixtures (*58*). Intuitively, this unique and high-performance gating behavior has motivated us to explore well-matched flexible MOFs or PCPs for C_3_F_6_/C_3_F_8_ separation.

**Fig. 1.**
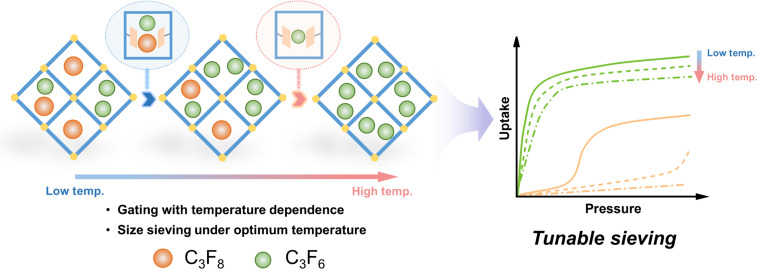
Illustration of the thermoregulatory gating effect facilitating the tunable molecular sieving of C_3_F_8_/C_3_F_6_ mixtures.

With this in mind, we here reported a microporous flexible-robust MOF, Ca-tcpb [denoted by **1**, H_4_tcpb = 1,2,4,5-tetrakis (4-carboxyphenyl) benzene], that features temperature-controllable gate opening, thus exhibiting a remarkable thermoregulatory gating effect for the sieving of C_3_F_8_/C_3_F_6_ mixtures under mild conditions. The structural flexibility of this MOF stems from the local twisting and vibrational motion of benzene rings (*R1*, *R2*, *R3*, *R4*) in the ligand. The temperature-driven sieving separation of linear/branched alkane isomers has demonstrated this flexibility (*46*). Unlike the double-door effect of fully flexible MOF for very similar molecular pairs (*59*), the desolvated Ca-tcpb (**1a**) remains porous for the smaller C_3_F_6_ at various temperatures. However, for the larger C_3_F_8_, it shows a temperature-responsive behavior, with the gating pressure increasing with temperature. This unique thermal response of the host-guest system is termed as “rigid-flexible combination (*60*).” Therefore, we can optimize the temperature to minimize the co-adsorption of the target gas and achieve efficient tunable molecular sieving (Fig. 1). As a result, **1a** displays a prominent C_3_F_6_ uptake (2.0 mmol g^−1^) but a negligible C_3_F_8_ adsorption (0.065 mmol g^−1^) at 298 K, placing it among the top porous materials. Breakthrough experiments show that it can produce high-purity C_3_F_8_ (over 99.999%) from a C_3_F_6_/C_3_F_8_ (10:90) mixture in one step, suggesting its potential for C_3_F_6_/C_3_F_8_ separation. C_3_F_6_-loaded single-crystal x-ray diffraction and theoretical calculations [dispersion-corrected density functional theory (DFT-D)] reveal the way of host-guest interactions. In situ powder x-ray diffraction (PXRD) further elucidates the adsorption mechanisms driven by C_3_F_6_ and C_3_F_8_ at the molecular level.

## RESULTS

**Fig. 2.**
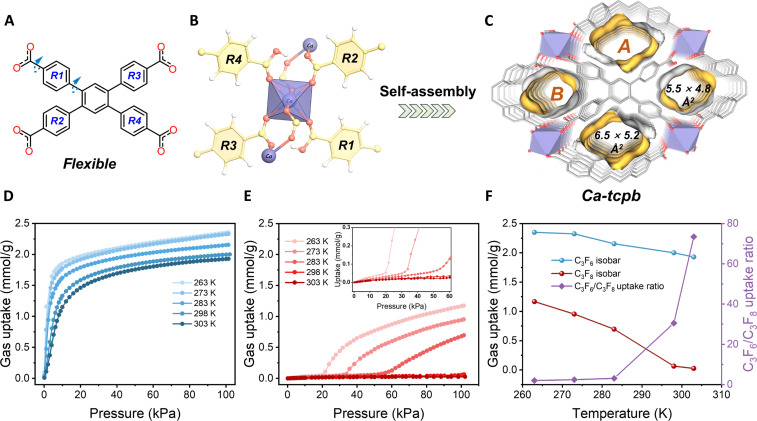
Structure and gas sorption properties of Ca-tcpb (1a). (**A**) Flexible nature of linker H_2_tcpb^2−^ (H atoms omitted); *R1*, *R2*, *R3*, and *R4* represent four rings of carboxyphenyl. (**B**) The local coordination environments of the H_2_tcpb^2−^ linker and calcium atoms. Ca (light purple), C (bright yellow), O (rosy red), and H (white). (**C**) Crystal structure of **1a** determined from single-crystal x-ray diffraction, showing one-dimensional channels within two types A/B. (**D**) C_3_F_6_ adsorption isotherms of **1a** at different temperatures. (**E**) C_3_F_8_ adsorption isotherms of **1a** at different temperatures. (**F**) Adsorption isobars (1 bar) of C_3_F_6_ (blue) and C_3_F_8_ (red), and the uptake ratio C_3_F_6_/C_3_F_8_ (purple) for **1a**.

The colorless prism crystals of **1** were obtained by a simple solvothermal reaction following the reported method (fig. S2) (*46*). The result of the single-crystal x-ray diffraction (SCXRD) revealed that **1** crystallizes in *P-1* space group, where one Ca ion and one dynamic H_2_tcpb^2−^ linker in its asymmetric units. Each octahedral Ca atom is coordinated by six O atoms of six different H_2_tcpb^2−^ linkers, forming two types of one-dimensional channels bridged by the organic linkers. Channels A and B have cross-sectional sizes of 6.5 × 5.2 Å^2^ and 5.5 × 4.8 Å^2^ on *bc* plane, respectively (without considering van der Waals radii) (Fig. 2, B and C, and fig. S3A). In addition, the partial deprotonation of the carboxyl groups results in channel B containing free-OH groups pointing to the pore centers. The hydrogen-rich channels A and B create a unique pore surface chemical environment. To investigate the structure-property relationships, we first calculated the electrostatic potential distributions of the channels and guests (C_3_F_6_ and C_3_F_8_). The mapping of electrostatic potential (figs. S3B and S4) shows that a positive potential trap (PPT) is generated due to the difference in polarizability between the carbon atoms and hydrogen atoms (C^δ^···H^δ+^), which also indicates propitious access to PPT and the possibility of multipoint interactions for C_3_F_6_ or C_3_F_8_ based on surface potential matching.

The PXRD pattern of **1** confirmed its bulk purity and matched the calculated pattern from the crystal structure (fig. S5). Thermogravimetric analysis (TGA) (fig. S6) and variable temperature PXRD (fig. S7) indicated that **1** is thermally stable up to approximately 400°C. The guest-free of **1** (1a) was prepared by activating **1** at 150°C under a high vacuum for 24 hours. The porosity of **1a** was verified by N_2_ adsorption/desorption isotherms at 77 K, which showed a stepwise adsorption behavior (fig. S8). The first step occurred at lower partial pressure (*P*/*P*_0_ = 0.007) with a N_2_ uptake of 47.6 cm^3^ g^−1^, followed by a gradual increase to 109.7 cm^3^ g^−1^ at higher relative pressure (0.1 < *P*/*P*_0_ < 1). The Brunauer-Emmett-Teller (BET) surface area of **1a** was calculated to be 260.65 m^2^ g^−1^, in agreement with the previously reported value. This extraordinary phenomenon indicated a flexible-robust feature of **1a**, which met the criteria of “flexible-robust” as define earlier, and also motivated us to assess the potential of **1a** for C_3_F_6_/C_3_F_8_ separation.

We then explored the ability of **1a** to separate C_3_F_6_ from C_3_F_8_ by measuring the single-component adsorption isotherms for both gases at various temperatures (263, 273, 283, 298, and 303 K). Figure 2 (D and E) shows that the isotherms of C_3_F_6_ on **1a** at all five temperatures were of type I profile, indicating a high affinity for C_3_F_6_. The C_3_F_6_ uptake on **1a** reached 1.34 mmol g^−1^ at 10 kPa and increased to 2.0 mmol g^−1^ at 298 K, 100 kPa. In contrast, C_3_F_8_ exhibited a stepwise adsorption behavior, suggesting a flexible response of the framework. The gate-opening pressure for C_3_F_8_ increased from 1.2 kPa (263 K) to around 20 kPa (273 K) and 42 kPa (283 K); with temperature, the step became less pronounced at higher pressures. As a result, the C_3_F_8_ adsorption on **1a** was negligible, only 0.065 mmol g^−1^ (1.3 cm^3^ g^−1^) at 298 K and 100 kPa. The uptake ratio curve (Fig. 2F) showed that these values could exceed 30 at temperatures above 298 K, implying an ultrahigh selectivity of **1a** for C_3_F_6_ over C_3_F_8_. To further elucidate the temperature-dependent C_3_F_8_ adsorption behavior of **1a**, we performed kinetic adsorption experiments on **1a** at different temperatures. Figure S9 shows that at 283 K and 100 kPa, there was a certain amount of C_3_F_8_ adsorption but slow diffusion equilibrium; however, at 40 kPa, which was below the gate-opening pressure, and at 298 K, 100 kPa, there was negligible C_3_F_8_ uptake consistent with Fig. 2E. This suggests that the diffusive rejection of C_3_F_8_ at near ambient pressure can be realized by simply altering the temperature. Thus, **1a** is a rare material that can use temperature-regulated gating pressure to enable C_3_F_6_/C_3_F_8_ sieving separation. This phenomenon for C_3_F_8_ is mainly ascribed to the temperature-induced guest response of the flexible framework. The gate-opening pressure of adsorption depends on both the binding affinity and molecular size of the guest. By investigating the relationship between C_3_F_8_ opening pressure and temperature (fig. S10A), it was found that there was a visible linear correlation (fig. S10B), which could not only determine the opening pressure of C_3_F_8_ at a specific temperature but also predict the optimal temperature corresponding to a favorable opening pressure (below the standard pressure) that could minimize the co-adsorption of C_3_F_8_. The anticipated results were consistent with the above experiments.

**Fig. 3.**
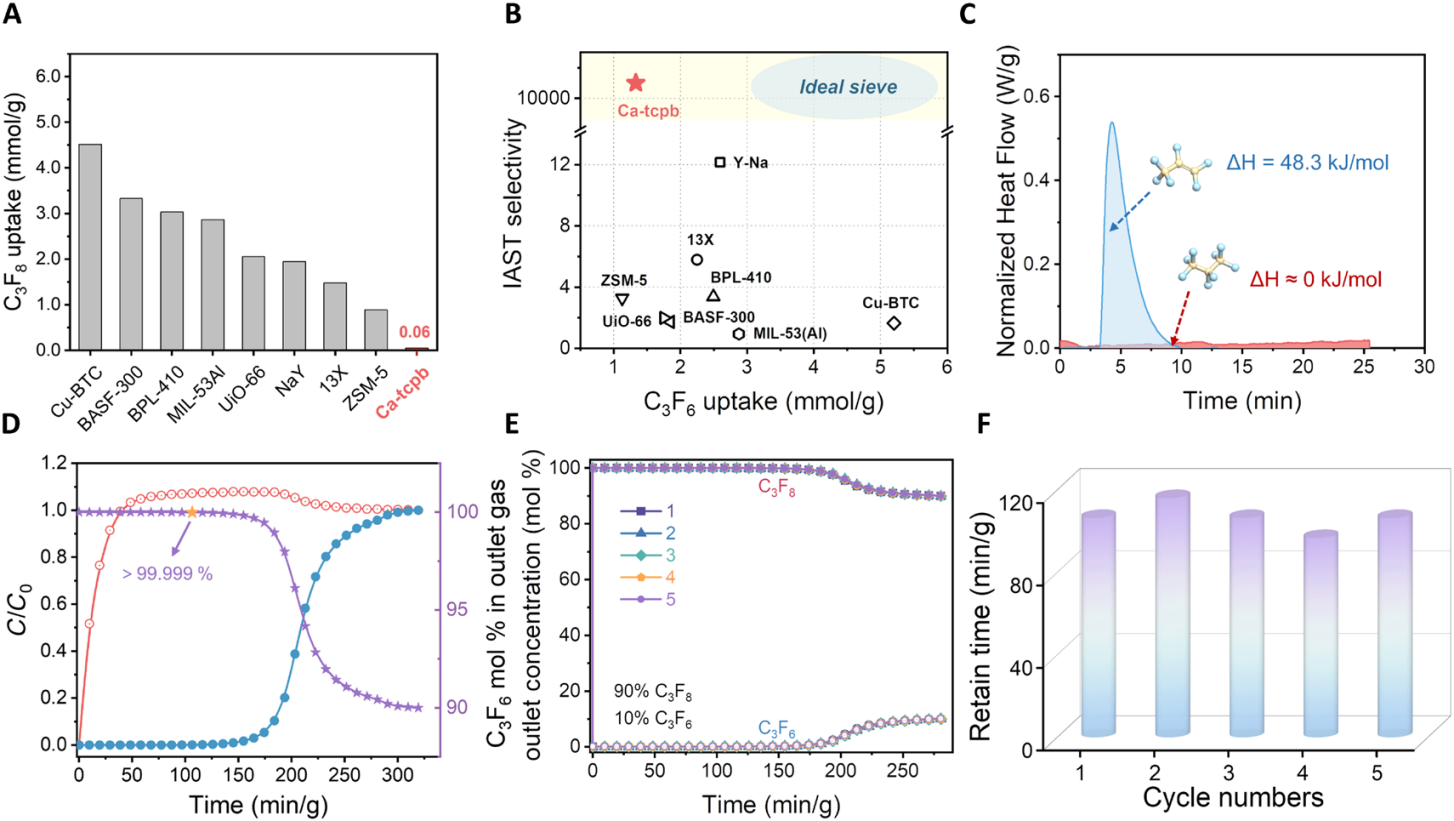
Separation performance and column breakthrough results of 1a. (**A**) C_3_F_8_ uptake of **1a** compared with other representative porous solids. (**B**) IAST adsorption selectivity (C_3_F_6_/C_3_F_8_, 10:90, v/v) and C_3_F_6_ uptake at 298 K and 0.1 bar for different porous solids. The ideal sieves are those that completely exclude C_3_F_8_ and adsorb large amounts of C_3_F_6_. (**C**) Heat of adsorption for C_3_F_6_ and C_3_F_8_ on **1a** obtained from Thermal gravimetric–Differential scanning calorimetry (TG-DSC) analysis. (**D** and **E**) Single and multicycle dynamic breakthrough curves of (10:90, v/v) C_3_F_6_/C_3_F_8_ gas mixtures using a packed column bed with **1a** at 298 K and 1 bar. The bright yellow pentagram in (D) indicates that the outlet purity of C_3_F_8_ is >99.999% at this point. (**F**) Retention time of the outlet concentration of C_3_F_8_ > 5N in five dynamic cycles.

To evaluate the separation performance of **1a** on C_3_F_6_/C_3_F_8_, we compared the isotherms of several well-known porous materials (e.g., zeolite and MOFs with OMSs) (fig. S11), which exhibited unsatisfactory results, primarily due to the co-adsorption of C_3_F_8_ and slow diffusion. In contrast, although **1a** had a modest BET area, its equilibrium adsorption of C_3_F_6_ (2.0 mmol g^−1^) was observably higher than the star molecular sieves like ZSM-5 (1.47 mmol g^−1^) and 13X (1.97 mmol g^−1^), but slightly lower than some MOFs, such as Cu-BTC (5.97 mmol g^−1^), BASF-300 (4.31 mmol g^−1^) with OMSs, UiO-66 (3.15 mmol g^−1^), and MIL-53Al (3.75 mmol g^−1^) with higher BET area and porosity. However, remarkably, as depicted in Fig. 3A, **1a** adsorbed only a negligible amount of C_3_F_8_ (0.06 mmol g^−1^), far less than all the porous materials mentioned above at 298 K, 100 kPa.

Next, we used ideal adsorbed solution theory (IAST) to calculate the selectivity and compare the separation potential of 10:90 (v/v) C_3_F_6_/C_3_F_8_ mixtures at 298 K (Fig. 3B and fig. S11S). **1a** showed an unprecedented selectivity of over 10,000, far higher than any others, including Cu-BTC (1.64), BASF-300 (1.72), MIL-53Al (~1.0), UiO-66 (1.98), BPL-410 (3.37), ZSM-5 (3.28), 13X (5.79), and NaY (12.2). Additionally, pure component isobar uptake ratio was also conducted to compare quantitatively. On the basis of the unique temperature gating effect, the ultralow C_3_F_8_ adsorption capacity of **1a** at 298 K resulted in an adsorption ratio of 30.5, the highest value among the porous materials described. This fascinating phenomenon provided a valuable platform for regulating the adsorption and selectivity of C_3_F_6_/C_3_F_8_, eventually achieving complete sieving of C_3_F_8_ from C_3_F_6_.

The enthalpy of adsorption represents the strength of the interaction between the C_3_F_6_/C_3_F_8_ molecules and **1a**, which plays a vital role in the separation. Differential scanning calorimetry of C_3_F_6_ and C_3_F_8_ adsorption was carried out on **1a** at 298 K and 1 bar. Figure 3C shows that this result further supported the complete exclusion of C_3_F_8_ from C_3_F_6_, as evidenced by the absence of a detectable heat of adsorption for C_3_F_8_, compared with the heat of sole adsorption for C_3_F_6_ of 48.3 kJ mol^−1^. Experimental adsorption enthalpies (*Q*_st_) were also obtained for the previously mentioned star materials to study the binding affinity to C_3_F_6_ (fig. S12). As depicted in fig. S12I, the *Q*_st_ of C_3_F_6_ on **1a** was higher than Cu-BTC (40.9 kJ mol^−1^), BASF-300 (31.8 kJ mol^−1^), MIL-53Al (47.4 kJ mol^−1^), UiO-66 (36.9 kJ mol^−1^), BPL-410 (48.0 kJ mol^−1^), and ZSM-5 (46.9 kJ mol^−1^) and second only to 13X (59.6 kJ mol^−1^) and NaY (53.5 kJ mol^−1^), demonstrating that **1a** had a strong thermodynamic affinity for the impurity C_3_F_6_, which was consistent with the steep adsorption isotherm. Remarkably, the moderate *Q_st_* value indicates reversible physisorption, and **1a** could be easily regenerated at 383 K for 10 to 20 min without losing its C_3_F_6_ adsorption capacity, as confirmed by 20 adsorption/desorption cycles (fig. S13).

The practical separation performance was evaluated with dynamic breakthrough experiments in which C_3_F_6_/C_3_F_8_ (v/v, 10:90) were fed over a packed column filled with **1a** at a flow rate of 1.5 cm^3^ min^−1^ under different temperatures. At 273 K, as the partial pressure of C_3_F_8_ in the mix gas exceeded the gating pressure threshold, the uptake of C_3_F_6_ was accompanied by the inevitable co-adsorption of C_3_F_8_. Thus, by increasing the opening pressure to 298 K to offset the co-adsorption of C_3_F_8_, as predicted (Fig. 3D and fig. S14), the complete separation of C_3_F_6_/C_3_F_8_ mixture could be efficiently realized, whereby C_3_F_8_ quickly eluted from the bed due to its negative adsorption capacity, followed by the preferential adsorption C_3_F_6_ at 106.5 min/g. On the basis of the breakthrough curve, the desired C_3_F_8_ was afforded directly from the production of 159.7 cm^3^ g^−1^ with a purity of over 99.999%, which attained the superior level (fig. S15A). In addition to high-purity yields, excellent repeatability is also essential to assess the economic and efficient separation potential of porous solids (Fig. 3E and fig. S15B). For five consecutive breakthrough cycles, **1a** could maintain the retention time of high-purity C_3_F_8_ (Fig. 3F), demonstrating its outstanding recyclability for C_3_F_6_/C_3_F_8_ separation. Notably, the activated material could preserve its initial high crystallinity and adsorption performance even after half a year of storage (fig. S16).

**Fig. 4.**
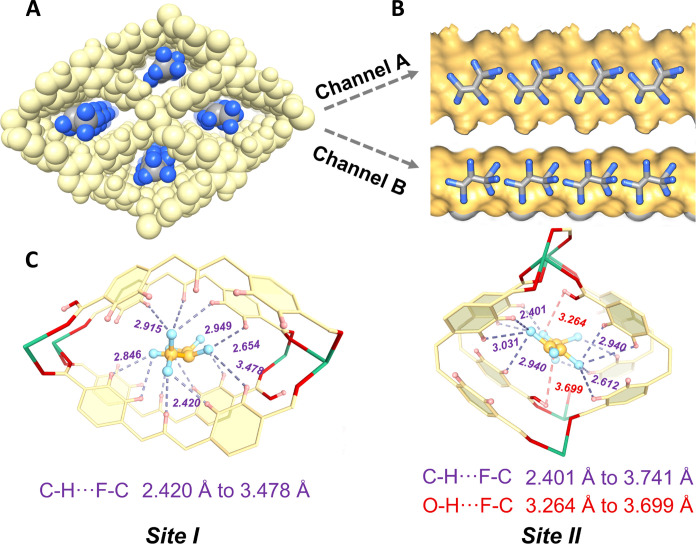
The binding sites in 1a. (**A**) Front and (**B**) side views of the packing diagram of the C_3_F_6_ adsorbed structure from SCXRD. The framework and pore surface are shown in pale gold and orange, light blue (F atoms), and gray spheres (C atoms). (**C**) Preferential binding sites for C_3_F_6_ molecules in channels (sites I and II) and valid interactions with the framework. Pale gold, blue-green, pink, red, and green represent the C, F, H, O, and Ca atoms, respectively.

To elucidate the interactions between C_3_F_6_ molecules and **1a** from a structural perspective as well as to determine the binding configuration of C_3_F_6_, we first performed single-crystal x-ray diffraction (SCXRD) measurements on C_3_F_6_-loaded **1a** at 170 K and DFT-D calculations to locate the C_3_F_6_ interaction sites in the channel of **1a** (Fig. 4, A and B). On the basis of these data, the location of C_3_F_6_ molecules was identified by notable increase in the residual electron. As shown in Fig. 4C, two preferential C_3_F_6_ adsorption sites were observed along the one-dimensional channels A and B. Multiple hydrogen bonding between the host and guest was annotated in detail, mainly C-F **···** H-C distances of 2.420 to 3.368 Å and C-F **···** H-O distances of 3.264 to 3.699 Å (only present in pore B due to the retention of free hydroxyl groups). The polarization between the electron-rich fluorine atoms of the guest molecule and the hydrogen atom of the benzene ring illustrates a strong host-guest binding interaction (*61*, *62*), which is also coherent with the adaptation of the PPT formed by the pore to match the electron-rich C_3_F_6_, as described earlier. Notably, the cell volume of **1a** undergoes expansion upon loading with C_3_F_6_. The lattice parameters of the resultant C_3_F_6_-filled phases were determined as follows: *a* = 5.1039(11) Å, *b* = 11.232(3) Å, *c* = 15.317(3) Å; α = 83.676(7)°, β = 88.420(7)°, γ = 83.908(7)°. The unit cell volume increases to 867.7(3) Å^3^, which is notably larger than the original **1a** volume of 838.56(6) Å^3^. This increase agrees with the expected occupation by gas molecules (fig. S17). In addition, *R1*, *R2*, *R3*, and *R4* all rotated to varying degrees after being loaded with C_3_F_6_ due to the rotation of the ligand tcpb carboxybenzene single bonds (C─C, C─O) (figs. S18 and S19), confirming the flexible nature of **1a**. Considering the structure changes after the adsorption of guests, the binding energy calculations were carried out with geometry optimization based on the guest molecule-loaded empty framework from SCXRD, which showed two effective sites, corresponding to the two types of channels of **1a**, with adsorption locations matching the above experiments (fig. S20). The calculated binding energies for C_3_F_6_ at sites I and II were 50.58 and 37.50 kJ mol^−1^, respectively. For C_3_F_8_, the calculated binding energies at sites I and II were 35.15 and 28.54 kJ mol^−1^, respectively. Moreover, molecular dynamics (MD) simulations demonstrated that C_3_F_8_ can only diffuse within the flexible host (*D* = 4.28 × 10^−7^ cm^2^ s^−1^) rather than the rigid host (*D* = 2.57 × 10^−8^ cm^2^ s^−1^) (fig. S21), indicating that transient pore opening is prerequisite for diffusion.

**Fig. 5.**
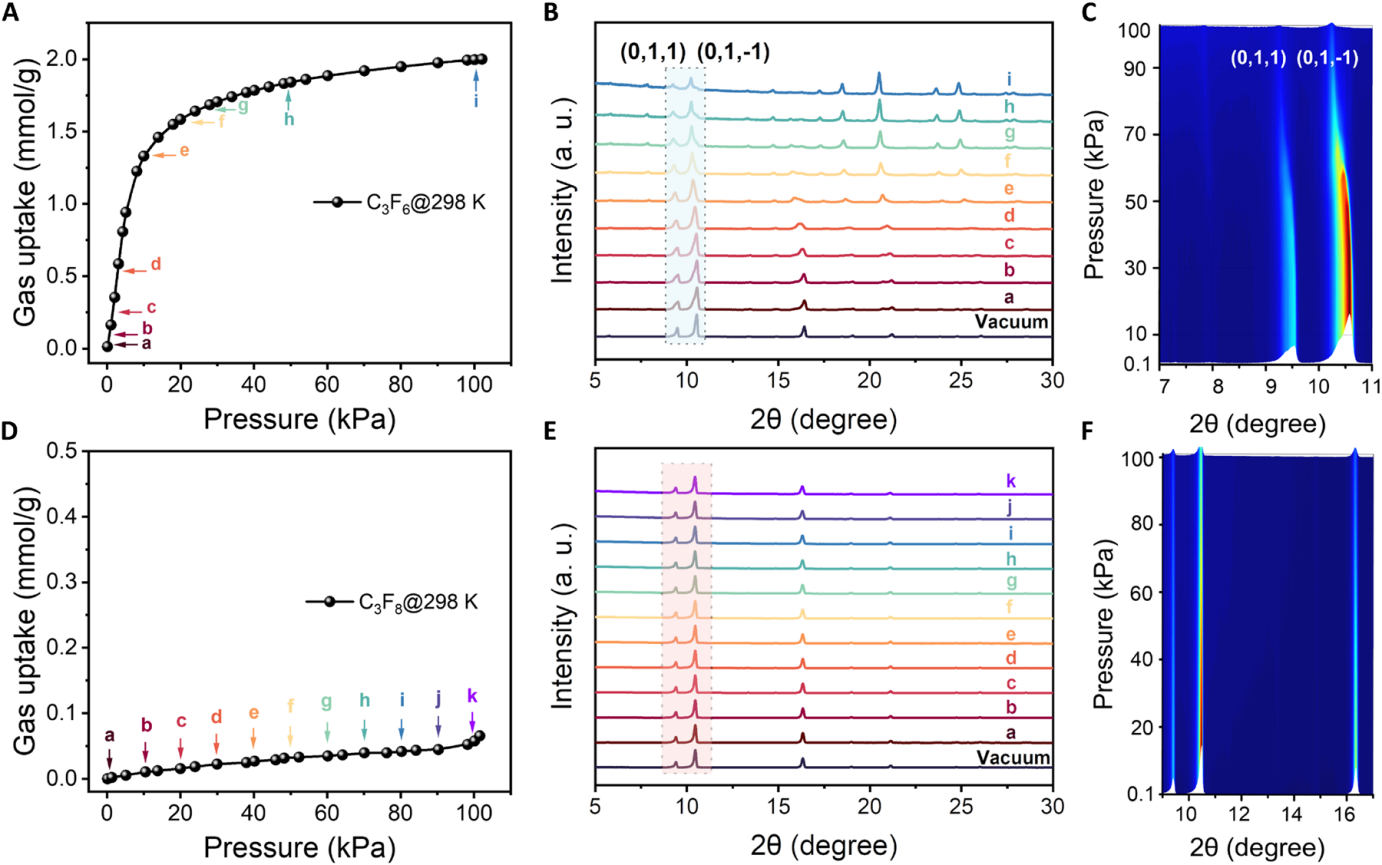
In situ guest-loaded PXRD at 298 K. Numbers of letters in adsorption correspond to the numbering of the PXRD patterns. (**A** and **D**) Adsorption isotherm of C_3_F_6_ and C_3_F_8_. (**B** and **E**) PXRD pattern recorded during loading C_3_F_6_ and C_3_F_8_ at different pressures. (**C** and **F**) Contour plot of the PXRD pattern evolution of C_3_F_6_ and C_3_F_8_ under different pressure.

To gain a deeper understanding of the “adsorption steps” behaviors with the flexible-robust **1a**, we collected in situ loading-dependent PXRD patterns for **1a** exposed to the atmosphere of C_3_F_6_ or C_3_F_8_ at relevant temperatures. When the framework adsorbs guest molecules, PXRD shows a shift in the peak or the emergence of new phases, corresponding to a slight structural transition associated with the expansion of the flexible channels. Figure 5 and fig. S22 show that for the loading of C_3_F_6_, at 283 and 298 K, a noticeable left shift ([0, 1, 1] and [0, 1, −1] crystal surface, consistent with unit cell expansion) with increasing pressure was observed, in agreement with the adsorption isotherm of C_3_F_6_ at these temperatures. PXRD patterns under the C_3_F_8_ atmosphere differed from those under C_3_F_6_. No obvious peak shift was observed at 298 K in Fig. 5 (D to F), suggesting no structural transformation due to the extremely low adsorption of C_3_F_8_. Nevertheless, at 283 K, the in situ PXRD patterns for C_3_F_8_ revealed two main phases, α and β, corresponding to the closed phase before gate opening and the open form after gate opening, respectively. Despite a small amount of C_3_F_8_ adsorption occurring just before isotherm inflection points, in situ PXRD data showed that pore opening only took place in a minimal or negligible portion (points a to f in the isotherm of C_3_F_8_). During progressive C_3_F_8_ gas loading (points g to l in the isotherm of C_3_F_8_), both α and β phases were present. Their relative proportions, inferred from the intensity of the powder diffraction peaks, correlated well with the relative amount adsorbed (fig. S25). Notably, some weak unidentified peaks emerged, which could be assigned to a forming metastable, distorted adsorbent structure induced by nonstoichiometric adsorption. This finding indicates that the stepwise sorption process was accompanied by an expansion of the pore window, indicative of a gate-opening effect. The gating pressure in the temperature-dependent pressure-gating sieving effect rises with increasing temperature. This, in turn, leads to a notable reduction in the adsorption of C_3_F_8_ by the material at temperatures exceeding 298 K. This phenomenon resulted from the combined effect of the guests and the external temperature. When there were no guests and only varying temperatures, the framework did not show substantial changes, as verified by the results for variable temperature single crystals of **1a** (fig. S26 and table S4). These above proofs indicate that for this rigid-flexible framework, regulating the gating pressure of the guests by optimizing the temperature effectively separates impurities from the product gas.

## DISCUSSION

In summary, we have successfully developed a flexible-robust microporous MOF (Ca-tcpb) with high sieving of C_3_F_8_/C_3_F_6_ separation by modulating the gate-opening pressure. At optimized temperature of 298 K and even at elevated temperatures, Ca-tcpb demonstrates a large capacity of C_3_F_6_ while completely excluding C_3_F_8_, outperforming several well-known porous materials tested. Moreover, it can achieve high purity (over 99.999%) and a remarkable recovery of C_3_F_8_ from 10:90 C_3_F_6_/C_3_F_8_ mixtures. These exceptional separation features are attributed to the temperature-dependent gating effect, which is corroborated by in situ PXRD analysis. This gating phenomenon provides valuable insights into the strategic utilization of adsorbent flexibility and host-guest interactions for the efficient separation of relevant industrial gas mixtures.

## MATERIALS AND METHODS

### Preparation of 1 and other porous solids

All reagents were used as received from commercial suppliers without further purification. Ca-tcpb was synthesized according to previously reported methods (*46*). Calcium chloride (CaCl_2_, ≥97%, Sigma-Aldrich), 1,2,4,5-tetrakis(4-carboxyphenyl) benzene (H_4_TCPB, Jilin ET Co. Ltd., 98%), and ethanol (Sinopharm Chemical Reagent Co. Ltd., GR) were used without further purification.

A mixture of CaCl_2_ (0.090 g) and H_4_TCPB (0.090 g) was dissolved in 10 ml of absolute ethanol and stirred for 2 hours. The reactants were then added to a 20-ml Teflon-lined autoclave and heated at 373 K for 72 hours. Upon cooling to room temperature, colorless prism crystals were obtained through filtration and washed with ethanol three times over 3 days. The details of other selected porous materials are shown in table S1.

### Sample characterization

PXRD patterns were recorded on a BRUKER D8 ADVANCE diffractometer using Cu-Kα radiation (λ = 0.1543 nm) at 40 kV from 4° to 60° (2θ angle range) with a step size of 0.02°. Single-crystal x-ray diffraction data collection and structural analysis of the crystals were performed at 170, 273, 293, and 313 K by using an Agilent Technologies Super Nova single-crystal diffractometer equipped with graphite monochromatic Cu-Kα radiation (λ = 1.54184 Å). Using Olex (*63*), the structure was solved with the ShelXT structure solution program using intrinsic phasing and refined by full matrix least-squares methods with the SHELXT program package. The thermal gravimetric analysis was carried out on a Simultaneous DSC/TGA TA instrument (SDT 650) analyzer with a ramping rate of 5°C/min under N_2_ atmosphere from 30° to 800°C.

### Single-crystal x-ray diffraction of C_3_F_6_-loaded 1a

The single crystal of **1** was immobilized inside a glass capillary and pretreated similarly to the gas adsorption measurement process to obtain the guest-free **1a**. After collecting single-crystal data for the guest-free **1a** in N_2_ atmosphere, the capillary with one open end was placed in a desiccator to backfill with C_3_F_6_ for 12 hours to obtain a gas-loaded single crystal. The desiccator was filled with C_3_F_6_ using Micromeritics ASAP 2020 PLUS HD88 surface area analyzer, and the pressure inside the desiccator was measured to be 1 atm. Subsequently, the capillary was sealed with plasticine and kept at a temperature of 170 K during the data collection process for C_3_F_6_-loaded **1a**. The detailed crystallographic data and structure refinement parameters for this compound are summarized in table S4.

### Variable temperature PXRD of 1a

Variable temperature PXRD patterns were measured on a PanAlytical X’pert PRO MRD x-ray diffractometer with Cu-Kα radiation (λ = 0.1543 nm) at 40 kV from 4° to 60°. PXRD patterns were recorded on pelletized (10 mm by 10 mm) as-made **1a** at various temperatures between 25° and 400°C with a heating rate of 5°C/min and a step of 0.02°.

### In situ gas-loaded PXRD of 1a

Data were recorded using a PANalytical X’Pert^3^ powder diffractometer equipped with a Cu sealed tube (λ = 1.54056 Å) at 40 kV and 40 mA. An Anton Paar TTK 600 stage coupled with the Anton Paar CCU 100 Temperature Control Unit was used to control the temperature. In a typical experiment, 10 mg of sample was activated and loaded on a zero-background sample holder made for the Anton Paar TTK 600 chamber. The sample was cooled from room temperature to 298 and 283 K by using liquid nitrogen set up for the Anton Paar TTK 600. The data were collected from 5° to 30° (2θ) with a step size of 0.0131303° and a scan time of 50 s per step. The PXRD patterns were recorded after equilibration (30 min) at selected points of the isotherm.

### Heats of adsorption calculations

Differential scanning calorimetry of C_3_F_6_ and C_3_F_8_ adsorption on activated samples of 5 to 10 mg at 298 K and 1 bar was used to calculate the isosteric enthalpy. The obtained signal is integrated to give the corresponding amount of heat in joules.

### Gas adsorption measurements

Hexafluoropropylene (C_3_F_6_, 99.999%), octafluoropropane (C_3_F_8_, 99.999%), sorption isotherms, and nitrogen adsorption (N_2_, 99.999%) at 77 K were measured by Micromeritics ASAP 2020 PLUS HD88 surface area analyzer. Around ~120 mg of as-prepared sample was activated under a dynamic vacuum at certain temperature before data collection. Kinetic sorption measurements were conducted by the Intelligent Gravimetric Analyzer (IGA001, Hiden, UK), which uses a gravimetric technique to accurately measure the gas adsorption on ~50-mg **1a** under the constant temperature and pressure conditions.

### Column breakthrough measurements

The Ca-tcpb was first activated under ultrahigh vacuum at 150°C for 24 hours. Column breakthrough tests were performed in a stainless-steel column (50 mm × 4.6 mm inside diameter) manually packed with activated samples **1a** (~0.310 g). Then, the column was swept at 100°C overnight with a helium flow. The binary mixtures C_3_F_6_/C_3_F_8_ (10:90) were carried out at a flow rate of 1.5 ml/min (273 and 298 K). The effluent from the column was analyzed using an online gas chromatography (GC-2010C, SHIMADZU) with FID. After the breakthrough tests, the column was regenerated with a helium flow at 100°C overnight.

### DFT calculations

All theoretical calculations were carried out under the generalized gradient approximation (GGA) with Perdew-Burke-Ernzerhof (PBE) in Material Studio 2019. The surface electrostatic potential of **1a**, C_3_F_6_, and C_3_F_8_ was calculated using the DMol^3^ modules. The energy, force, and displacement convergence criteria were set as 1 × 10^−5^ Ha, 0.002 Ha/Å, and 0.005 Å, respectively. The electron density data obtained from these calculations were used to construct the 0.017 e^−^ Å^−3^ electron density isosurfaces of C_3_F_6_ and C_3_F_8_, while the electron density data of both frameworks were used to construct the 0.2 e^−^ Å^−3^ electron density isosurfaces. The calculated electrostatic potential for **1a** and C_3_F_6_ and C_3_F_8_ was then mapped onto their electron density isosurfaces. The binding energy between the guests and framework was performed with the CASTEP package. A cutoff energy of 544 eV and the 4 × 2 × 2 *k*-point were set to be enough for the total energy to converge within 0.01 meV/atom. The structure of the activated framework and guests was optimized with the same parameters to obtain the minimal energy. Then, the guests were then placed to different locations of the channel with a full structural relaxation. The host-guest binding energy was determined by the following equation: *E*_B_ = *E*_(guest)_ + *E*_(adsorbent)_ − *E*_(adsorbent+guest)_. MD simulations were conducted using the isothermal-isobaric ensemble with constant pressure/temperature, employing a Nose thermostat and random initial velocities. The Ewald summation method was used to calculate both Van der Waals interactions and electrostatic interactions. All buffer widths were set as 0.5 Å. A time step of 1.0 fs was used, and the total simulation time was established at 1000 ps under 283 K. Electrostatic and charge equilibration charges were applied to the host structure and guest molecules, respectively.

## Supplementary Materials

### This PDF file includes:

Supplementary text Figs. S1 to S26 Tables S1 to S5 References

## References

[1] J. R. Clark, Chemistry of electronic gases. J. Chem. Educ. 83, 857 (2006).

[2] T. Alsop, Semiconductor industry sales worldwide 1987–2022 (2023); https://www.statista.com.

[3] M. B. Chang, J. S. Chang, Abatement of PFCs from semiconductor manufacturing processes by nonthermal plasma technologies: A critical review. Ind. Eng. Chem. Res. 45, 4101–4109 (2006).

[4] H. M. Lee, S. H. Chen, Thermal abatement of perfluorocompounds with plasma torches. Energy Procedia 142, 3637–3643 (2017).

[5] W. T. Tsai, H. P. Chen, W. Y. Hsien, A review of uses, environmental hazards and recovery/recycle technologies of perfluorocarbons (PFCs) emissions from the semiconductor manufacturing processes. J. Loss Prev. Process Ind. 15, 65–75 (2002).

[6] A. J. Sicard, R. T. Baker, Fluorocarbon refrigerants and their syntheses: Past to present. Chem. Rev. 120, 9164–9303 (2020). 32809811 10.1021/acs.chemrev.9b00719

[7] R. T. Yang, *Gas Separation by Adsorption Processes* (World Scientific, 1997), vol. 1.

[8] D. S. Sholl, R. P. Lively, Seven chemical separations to change the world. Nature 532, 435–437 (2016). 27121824 10.1038/532435a

[9] M. B. Shiflett, D. R. Corbin, B. A. Elliott, S. Subramoney, K. Kaneko, A. Yokozeki, Sorption of trifluoromethane in activated carbon. Adsorption 20, 565–575 (2014).

[10] X. Huang, F. Chen, H. Sun, W. Xia, Z. Zhang, Q. Yang, Y. Yang, Q. Ren, Z. Bao, Separation of perfluorinated electron specialty gases on microporous carbon adsorbents with record selectivity. Sep. Purif. Technol. 292, 121059 (2022).

[11] D. Cao, S. Sircar, Heat of adsorption of pure sulfur hexafluoride on micro-mesoporous adsorbents. Adsorption 7, 73–80 (2001).

[12] J. Dunne, M. Rao, S. Sircar, R. Gorte, A. Myers, Calorimetric heats of adsorption and adsorption isotherms. 2. O_2_, N_2_, Ar, CO_2_, CH_4_, C_2_H_6_, and SF6 on NaX, H-ZSM-5, and Na-ZSM-5 zeolites. Langmuir 12, 5896–5904 (1996).

[13] I. M. Martos, J. Á. Ossorio, J. G. Sevillano, M. Doblaré, A. M. Calvo, S. Calero, Zeolites for the selective adsorption of sulfur hexafluoride. Phys. Chem. Chem. Phys. 17, 18121–18130 (2015). 26099734 10.1039/c5cp02407b

[14] H. C. Zhou, J. R. Long, O. M. Yaghi, Introduction to metal-organic frameworks. Chem. Rev. 112, 673–674 (2012). 22280456 10.1021/cr300014x

[15] H. Furukawa, K. E. Cordova, M. O’Keeffe, O. M. Yaghi, The chemistry and applications of metal-organic frameworks. Science 341, 1230444 (2013). 23990564 10.1126/science.1230444

[16] S. Kitagawa, R. Kitaura, S.-I. Noro, Functional porous coordination polymers. Angew. Chem. Int. Ed. Engl. 43, 2334–2375 (2004). 15114565 10.1002/anie.200300610

[17] X. Zhao, Y. Wang, D. S. Li, X. Bu, P. Feng, Metal-organic frameworks for separation. Adv. Mater. 30, 1705189 (2018). 10.1002/adma.201705189 29582482

[18] J. R. Li, R. J. Kuppler, H. C. Zhou, Selective gas adsorption and separation in metal-organic frameworks. Chem. Soc. Rev. 38, 1477–1504 (2009). 19384449 10.1039/b802426j

[19] B. Moulton, M. J. Zaworotko, From molecules to crystal engineering: Supramolecular isomerism and polymorphism in network solids. Chem. Rev. 101, 1629–1658 (2001). 11709994 10.1021/cr9900432

[20] D. K. Wanigarathna, J. Gao, B. Liu, Metal organic frameworks for adsorption-based separation of fluorocompounds: A review. Mater. Adv. 1, 310–320 (2020).

[21] M. E. Zick, J. H. Lee, M. I. Gonzalez, E. O. Velasquez, A. A. Uliana, J. Kim, J. R. Long, P. J. Milner, Fluoroarene separations in metal-organic frameworks with two proximal Mg^2+^ coordination sites. J. Am. Chem. Soc. 143, 1948–1958 (2021). 33492140 10.1021/jacs.0c11530 PMC8224537

[22] P. J. Kim, Y. W. You, H. Park, J. S. Chang, Y. S. Bae, C. H. Lee, J. K. Suh, Separation of SF_6_ from SF_6_/N_2_ mixture using metal-organic framework MIL-100(Fe) granule. Chem. Eng. J. 262, 683–690 (2015).

[23] J. Zheng, R. S. Vemuri, L. Estevez, P. K. Koech, T. Varga, D. M. Camaioni, T. A. Blake, B. P. McGrail, R. K. Motkuri, Pore-engineered metal-organic frameworks with excellent adsorption of water and fluorocarbon refrigerant for cooling applications. J. Am. Chem. Soc. 139, 10601–10604 (2017). 28702994 10.1021/jacs.7b04872

[24] M. B. Kim, S. J. Lee, C. Y. Lee, Y. S. Bae, High SF_6_ selectivities and capacities in isostructural metal-organic frameworks with proper pore sizes and highly dense unsaturated metal sites. Microporous Mesoporous Mater. 190, 356–361 (2014).

[25] J. Zheng, D. Barpaga, B. A. Trump, M. Shetty, Y. Fan, P. Bhattacharya, J. J. Jenks, C. Y. Su, C. M. Brown, G. Maurin, B. P. McGrail, R. K. Motkuri, Molecular insight into fluorocarbon adsorption in pore expanded metal-organic framework analogs. J. Am. Chem. Soc. 142, 3002–3012 (2020). 31968934 10.1021/jacs.9b11963 PMC11060419

[26] R. K. Motkuri, H. V. Annapureddy, M. Vijaykumar, H. T. Schaef, P. F. Martin, B. P. McGrail, L. X. Dang, R. Krishna, P. K. Thallapally, Fluorocarbon adsorption in hierarchical porous frameworks. Nat. Commun. 5, 4368 (2014). 25006832 10.1038/ncomms5368

[27] M. I. Hashim, H. T. Le, T. H. Chen, Y. S. Chen, O. Daugulis, C. W. Hsu, A. J. Jacobson, W. Kaveevivitchai, X. Liang, T. Makarenko, O. Š. Miljanić, I. Popovs, H. V. Tran, X. Wang, C. Wu, J. I. Wu, Dissecting porosity in molecular crystals: Influence of geometry, hydrogen bonding, and [π···π] stacking on the solid-state packing of fluorinated aromatics. J. Am. Chem. Soc. 140, 6014–6026 (2018). 29656637 10.1021/jacs.8b02869

[28] T. H. Chen, I. Popov, W. Kaveevivitchai, Y. C. Chuang, Y. S. Chen, O. Daugulis, A. J. Jacobson, O. Š. Miljanić, Thermally robust and porous noncovalent organic framework with high affinity for fluorocarbons and CFCs. Nat. Commun. 5, 5131 (2014). 25307413 10.1038/ncomms6131

[29] T. H. Chen, I. Popov, W. Kaveevivitchai, Y. C. Chuang, Y. S. Chen, A. J. Jacobson, O. Š. Miljanić, Mesoporous fluorinated metal-organic frameworks with exceptional adsorption of fluorocarbons and CFCs. Angew. Chem. Int. Ed. Engl. 54, 13902–13906 (2015). 26423312 10.1002/anie.201505149

[30] H. Wang, L. Yu, Y. Lin, J. Peng, S. J. Teat, L. J. Williams, J. Li, Adsorption of fluorocarbons and chlorocarbons by highly porous and robust fluorinated zirconium metal-organic frameworks. Inorg. Chem. 59, 4167–4171 (2020). 32186862 10.1021/acs.inorgchem.0c00018

[31] J. Y. S. Lin, Molecular sieves for gas separation. Science 353, 121–122 (2016). 27387937 10.1126/science.aag2267

[32] Y. Wang, D. Zhao, Beyond equilibrium: Metal-organic frameworks for molecular sieving and kinetic gas separation. Cryst. Growth Des. 17, 2291–2308 (2017).

[33] T. L. Hu, H. Wang, B. Li, R. Krishna, H. Wu, W. Zhou, Y. Zhao, Y. Han, X. Wang, W. Zhu, Z. Yao, S. Xiang, B. Chen, Microporous metal-organic framework with dual functionalities for highly efficient removal of acetylene from ethylene/acetylene mixtures. Nat. Commun. 6, 7328 (2015). 26041691 10.1038/ncomms8328 PMC4468854

[34] B. Li, X. Cui, D. O'Nolan, H. M. Wen, M. Jiang, R. Krishna, H. Wu, R. B. Lin, Y. S. Chen, D. Yuan, H. Xing, W. Zhou, Q. Ren, G. Qian, M. J. Zaworotko, B. Chen, An ideal molecular sieve for acetylene removal from ethylene with record selectivity and productivity. Adv. Mater. 29, 1704210 (2017). 10.1002/adma.201704210 29125651

[35] Z. Bao, J. Wang, Z. Zhang, H. Xing, Q. Yang, Y. Yang, H. Wu, R. Krishna, W. Zhou, B. Chen, Q. Ren, Molecular sieving of ethane from ethylene through the molecular cross-section size differentiation in gallate-based metal-organic frameworks. Angew. Chem. Int. Ed. Engl. 57, 16020–16025 (2018). 30304568 10.1002/anie.201808716 PMC7954079

[36] R. B. Lin, L. Li, H. L. Zhou, H. Wu, C. He, S. Li, R. Krishna, J. Li, W. Zhou, B. Chen, Molecular sieving of ethylene from ethane using a rigid metal-organic framework. Nat. Mater. 17, 1128–1133 (2018). 30397312 10.1038/s41563-018-0206-2

[37] T. Ke, Q. Wang, J. Shen, J. Zhou, Z. Bao, Q. Yang, Q. Ren, Molecular sieving of C2‐C3 Alkene from alkyne with tuned threshold pressure in robust layered metal-organic frameworks. Angew. Chem. Int. Ed. Engl. 59, 12725–12730 (2020). 32329164 10.1002/anie.202003421

[38] A. Cadiau, K. Adil, P. M. Bhatt, Y. Belmabkhout, M. Eddaoudi, A metal-organic framework-based splitter for separating propylene from propane. Science 353, 137–140 (2016). 27387945 10.1126/science.aaf6323

[39] H. Wang, X. Dong, V. Colombo, Q. Wang, Y. Liu, W. Liu, X. L. Wang, X. Y. Huang, D. M. Proserpio, A. Sironi, Y. Han, J. Li, Tailor-made microporous metal-organic frameworks for the full separation of propane from propylene through selective size exclusion. Adv. Mater. 30, e1805088 (2018). 30368929 10.1002/adma.201805088

[40] L. Yu, X. Han, H. Wang, S. Ullah, Q. Xia, W. Li, J. Li, I. Da Silva, P. Manuel, S. Rudić, Y. Cheng, S. Yang, T. Thonhauser, J. Li, Pore distortion in a metal-organic framework for regulated separation of propane and propylene. J. Am. Chem. Soc. 143, 19300–19305 (2021). 34780153 10.1021/jacs.1c10423

[41] Y. Xie, Y. Shi, E. M. Cedeño Morales, A. El Karch, B. Wang, H. Arman, K. Tan, B. Chen, Optimal binding affinity for sieving separation of propylene from propane in an oxyfluoride anion-based metal-organic framework. J. Am. Chem. Soc. 145, 2386–2394 (2023). 36691701 10.1021/jacs.2c11365

[42] B. Liang, X. Zhang, Y. Xie, R. B. Lin, R. Krishna, H. Cui, Z. Li, Y. Shi, H. Wu, W. Zhou, B. Chen, An ultramicroporous metal-organic framework for high sieving separation of propylene from propane. J. Am. Chem. Soc. 142, 17795–17801 (2020). 32991159 10.1021/jacs.0c09466 PMC10493866

[43] Q. Dong, Y. Huang, J. Wan, Z. Lu, Z. Wang, C. Gu, J. Duan, J. Bai, Confining water nanotubes in a Cu_10_O_13_-based metal-organic framework for propylene/propane separation with record-high selectivity. J. Am. Chem. Soc. 145, 8043–8051 (2023). 36995302 10.1021/jacs.3c00515

[44] S. Jiang, H. Sun, K. Gong, X. Huang, Y. Zhu, X. Feng, J. Xie, J. Liu, B. Wang, Metal-organic frameworks for breakthrough separation of 2-butene isomers with high dynamic selectivity and capacity. Angew. Chem. Int. Ed. Engl. 62, e202302036 (2023). 36950947 10.1002/anie.202302036

[45] A. H. Assen, Y. Belmabkhout, K. Adil, P. M. Bhatt, D. X. Xue, H. Jiang, M. Eddaoudi, Ultra-tuning of the rare-earth fcu-MOF aperture size for selective molecular exclusion of branched paraffins. Angew. Chem. Int. Ed. Engl. 54, 14353–14358 (2015). 26429515 10.1002/anie.201506345

[46] H. Wang, X. Dong, E. Velasco, D. H. Olson, Y. Han, J. Li, One-of-a-kind: A microporous metal-organic framework capable of adsorptive separation of linear, mono- and di-branched alkane isomers via temperature- and adsorbate-dependent molecular sieving. Energy Environ. Sci. 11, 1226–1231 (2018).

[47] D. D. Zhou, P. Chen, C. Wang, S. S. Wang, Y. Du, H. Yan, Z. M. Ye, C. T. He, R. K. Huang, Z. W. Mo, N. Y. Huang, J. P. Zhang, Intermediate-sized molecular sieving of styrene from larger and smaller analogues. Nat. Mater. 18, 994–998 (2019). 31308517 10.1038/s41563-019-0427-z

[48] L. Li, L. Guo, D. H. Olson, S. Xian, Z. Zhang, Q. Yang, K. Wu, Y. Yang, Z. Bao, Q. Ren, J. Li, Discrimination of xylene isomers in a stacked coordination polymer. Science 377, 335–339 (2022). 35857587 10.1126/science.abj7659

[49] D. O'Hagan, Understanding organofluorine chemistry. An introduction to the C-F bond. Chem. Soc. Rev. 37, 308–319 (2008). 18197347 10.1039/b711844a

[50] S. Mohanty, A. V. McCormick, Prospects for principles of size and shape selective separations using zeolites. Chem. Eng. J. 74, 1–14 (1999).

[51] X. W. Zhang, D. D. Zhou, J. P. Zhang, Tuning the gating energy barrier of metal-organic framework for molecular sieving. Chem 7, 1006–1019 (2021).

[52] D. Tanaka, K. Nakagawa, M. Higuchi, S. Horike, Y. Kubota, T. C. Kobayashi, M. Takata, S. Kitagawa, Kinetic gate-opening process in a flexible porous coordination polymer. Angew. Chem. Int. Ed. Engl. 47, 3914–3918 (2008). 18404764 10.1002/anie.200705822

[53] L. Bondorf, J. L. Fiorio, V. Bon, L. Zhang, M. Maliuta, S. Ehrling, I. Senkovska, J. D. Evans, J. O. Joswig, S. Kaskel, T. Heine, M. Hirscher, Isotope-selective pore opening in a flexible metal-organic framework. Sci. Adv. 8, eabn7035 (2022). 35417239 10.1126/sciadv.abn7035 PMC9007508

[54] C. Gu, N. Hosono, J. J. Zheng, Y. Sato, S. Kusaka, S. Sakaki, S. Kitagawa, Design and control of gas diffusion process in a nanoporous soft crystal. Science 363, 387–391 (2019). 30679369 10.1126/science.aar6833

[55] H. Zeng, M. Xie, T. Wang, R. J. Wei, X. J. Xie, Y. Zhao, W. Lu, D. Li, Orthogonal-array dynamic molecular sieving of propylene/propane mixtures. Nature 595, 542–548 (2021). 34290429 10.1038/s41586-021-03627-8

[56] Y. Chen, Y. Yang, Y. Wang, Q. Xiong, J. Yang, S. Xiang, L. Li, J. Li, Z. Zhang, B. Chen, Ultramicroporous hydrogen-bonded organic framework material with a thermoregulatory gating effect for record propylene separation. J. Am. Chem. Soc. 144, 17033–17040 (2022). 36069372 10.1021/jacs.2c06585

[57] Y. Yang, L. Li, R. B. Lin, Y. Ye, Z. Yao, L. Yang, F. Xiang, S. Chen, Z. Zhang, S. Xiang, B. Chen, Ethylene/ethane separation in a stable hydrogen-bonded organic framework through a gating mechanism. Nat. Chem. 13, 933–939 (2021). 34239085 10.1038/s41557-021-00740-z

[58] Q. Dong, X. Zhang, S. Liu, R. B. Lin, Y. Guo, Y. Ma, A. Yonezu, R. Krishna, G. Liu, J. Duan, R. Matsuda, W. Jin, B. Chen, Tuning gate-opening of a flexible metal-organic framework for ternary gas sieving separation. Angew. Chem. Int. Ed. Engl. 59, 22756–22762 (2020). 32876973 10.1002/anie.202011802

[59] X. Wang, R. Krishna, L. Li, B. Wang, T. He, Y. Z. Zhang, J. R. Li, J. Li, Guest-dependent pressure induced gate-opening effect enables effective separation of propene and propane in a flexible MOF. Chem. Eng. J. 346, 489–496 (2018).

[60] L. Li, R. B. Lin, R. Krishna, X. Wang, B. Li, H. Wu, J. Li, W. Zhou, B. Chen, Flexible-robust metal-organic framework for efficient removal of propyne from propylene. J. Am. Chem. Soc. 139, 7733–7736 (2017). 28580788 10.1021/jacs.7b04268

[61] J. L. Atwood, L. J. Barbour, A. Jerga, Storage of methane and freon by interstitial van der Waals confinement. Science 296, 2367–2369 (2002). 12004074 10.1126/science.1072252

[62] S. M. Wang, X. T. Mu, H. R. Liu, S. T. Zheng, Q. Y. Yang, Pore-structure control in metal-organic frameworks (MOFs) for capture of the greenhouse gas SF_6_ with record separation. Angew. Chem. Int. Ed. Engl. 61, e202207066 (2022). 35674195 10.1002/anie.202207066

[63] G. M. Sheldrick, SHELXT-Integrated space-group and crystal-structure determination. Acta Crystallogr. A Found. Adv. 71 (Pt 1), 3–8 (2015). 25537383 10.1107/S2053273314026370 PMC4283466

[64] P. S. Bárcia, D. Guimarães, P. A. P. Mendes, J. A. C. Silva, V. Guillerm, H. Chevreau, C. Serre, A. E. Rodrigues, Reverse shape selectivity in the adsorption of hexane and xylene isomers in MOF UiO-66. Microporous Mesoporous Mater. 139, 67–73 (2011).

[65] F. Xu, Y. Yu, J. Yan, Q. Xia, H. Wang, J. Li, Z. Li, Ultrafast room temperature synthesis of GrO@HKUST-1 composites with high CO_2_ adsorption capacity and CO_2_/N_2_ adsorption selectivity. Chem. Eng. J. 303, 231–237 (2016).

[66] H. R. Abid, Z. H. Rada, J. Shang, S. Wang, Synthesis, characterization, and CO_2_ adsorption of three metal-organic frameworks (MOFs): MIL-53, MIL-96, and amino-MIL-53. Polyhedron 120, 103–111 (2016).

