# Supplementary material for: Temperature-dependent molecular sieving of fluorinated propane/propylene mixtures by a flexible-robust metal-organic framework
Source: Sci Adv. 2024 Jan 19;10(3):eadj6473. doi: 10.1126/sciadv.adj6473 (PMC10798556; doi:10.1126/sciadv.adj6473)
Supplement: Supplementary file 1 — Supplementary text Figs. S1 to S26 Tables S1 to S5 References [file sciadv.adj6473_sm.pdf]

Science Advances
AAAS

Supplementary Materials for

Temperature-dependent molecular sieving of fluorinated propane/propylene mixtures by a flexible-robust metal-organic framework

Wei Xia *et al.*

Corresponding author: Lihang Chen, chenlihang@zju.edu.cn; Zhangjing Zhang, zzhang@fjnu.edu.cn; Zongbi Bao, baozb@zju.edu.cn

Sci. Adv. **10**, eadj6473 (2024)
DOI: 10.1126/sciadv.adj6473

This PDF file includes:

Supplementary text
Figs. S1 to S26
Tables S1 to S5
References

Fitting of pure component isotherms

The experimentally measured loadings for C_3F_6 and C_3F_8 measured at temperatures of 298 K in selected material were fitted with the Dual-Langmuir-Freundlich isotherm model.

$$q = q_{A,sat}\frac{b_A p^{n_A}}{1 + b_A p^{n_A}} + q_{B,sat}\frac{b_B p^{n_B}}{1 + b_B p^{n_B}}$$

where $q_{A,sat}$ and $q_{B,sat}$ (mol·kg^{-1}) are the saturated capacities of sites A and B, respectively, b_A and b_B (1/kPa) are the affinity coefficients to the sites A and B, respectively, p (kPa) is the pressure of the bulk gas at equilibrium with the adsorbed phase (kPa), q (mol·kg^{-1}) is the gas uptake amount of an adsorbent, and n_A and n_B represent the deviations from an ideal homogeneous surface.

IAST calculations of adsorption selectivity

In order to compare the C_3F_6/C_3F_8 separation potential of various porous materials, IAST calculations of mixture adsorption (10/90) were performed. For separation of a binary mixture of components i and j, the selectivity coefficient (S_{ij}) has been defined as:

$$S_{ij} = \frac{q_i/y_i}{q_j/y_j}$$

Where, q_i and q_j refer to the equilibrated adsorption capacity of component i and j, y_i and y_j refer to the molar fraction of component i and j in gas phase.

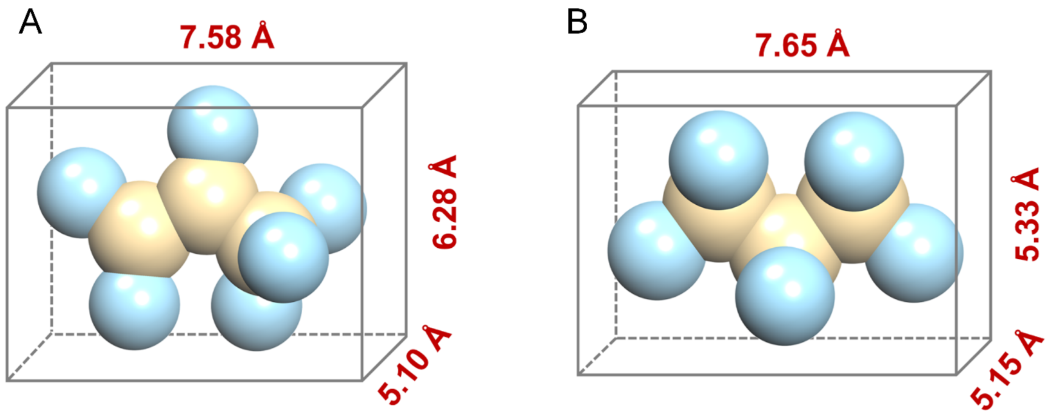

Fig. S1. Molecular dimensions of guests: (**A**) C_3F_6 and (**B**) C_3F_8.

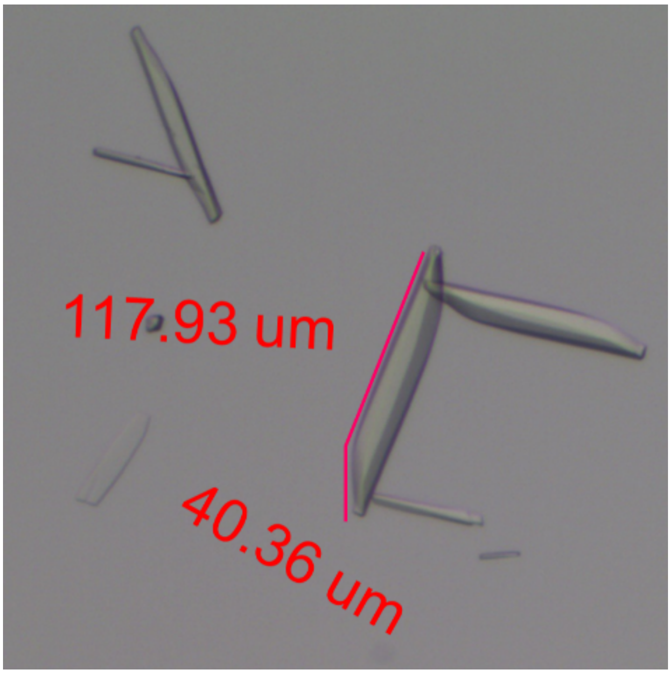

Fig. S2. The photoshoot of the as-synthesized crystals of Ca-tcpb.

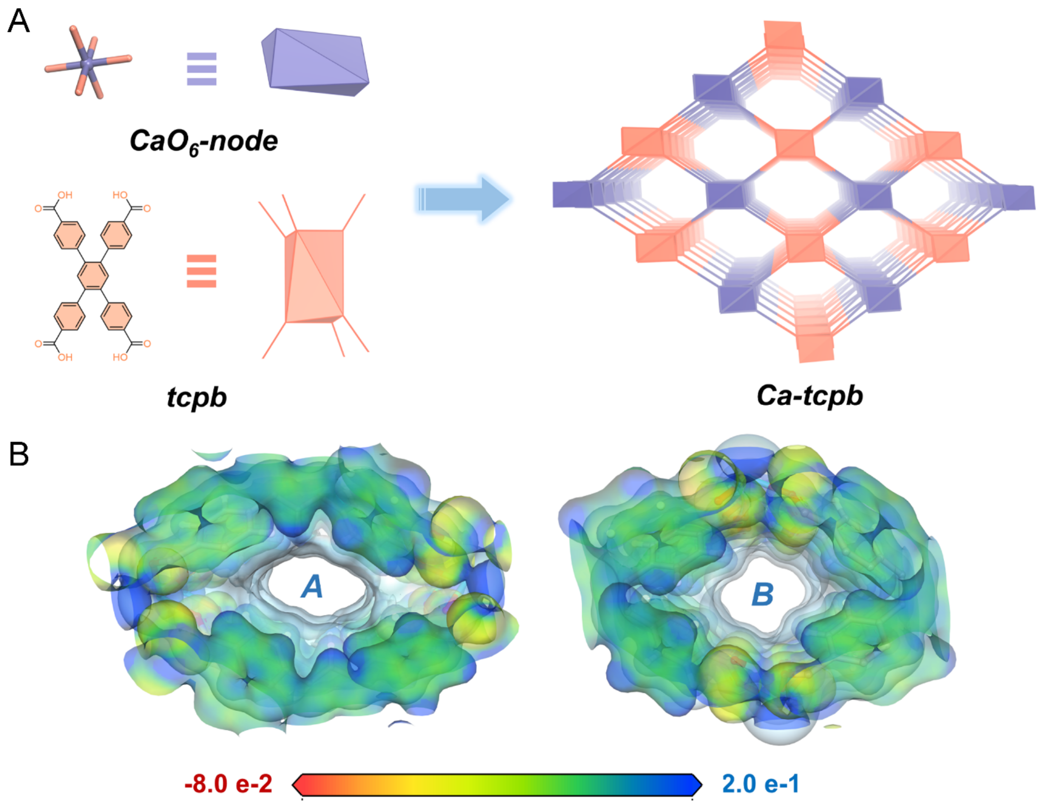

Fig. S3. Structure analysis and electrostatic potential calculation of 1a. (**A**) Inorganic and organic building units, crystal structure, and topology of Ca-tcpb. (**B**) The surface electrostatic potential of **1a** mapped onto the 0.01 Hartree/e density isosurface with a scale spanning from -0.08 Hartree/e (red) through 0 to 0.2 Hartree/e (blue).

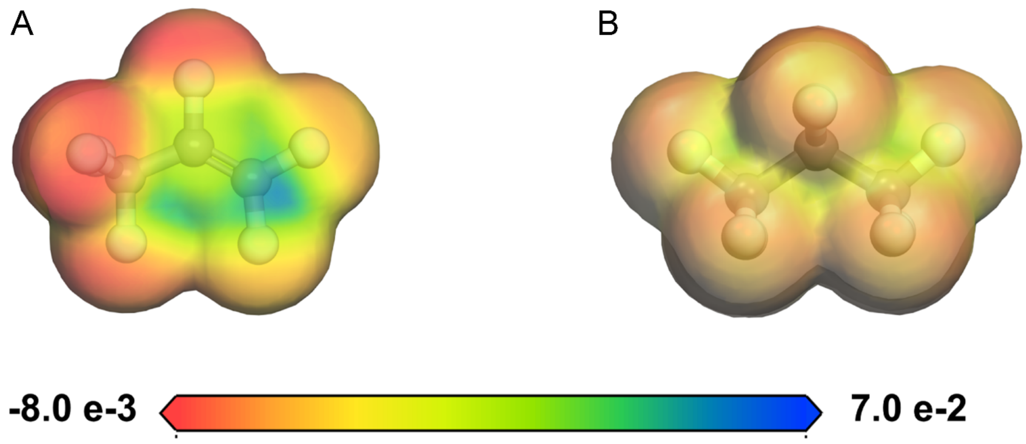

Fig. S4. The surface electrostatic potential of guests: (**A**) C_3F_6 and (**B**) C_3F_8.

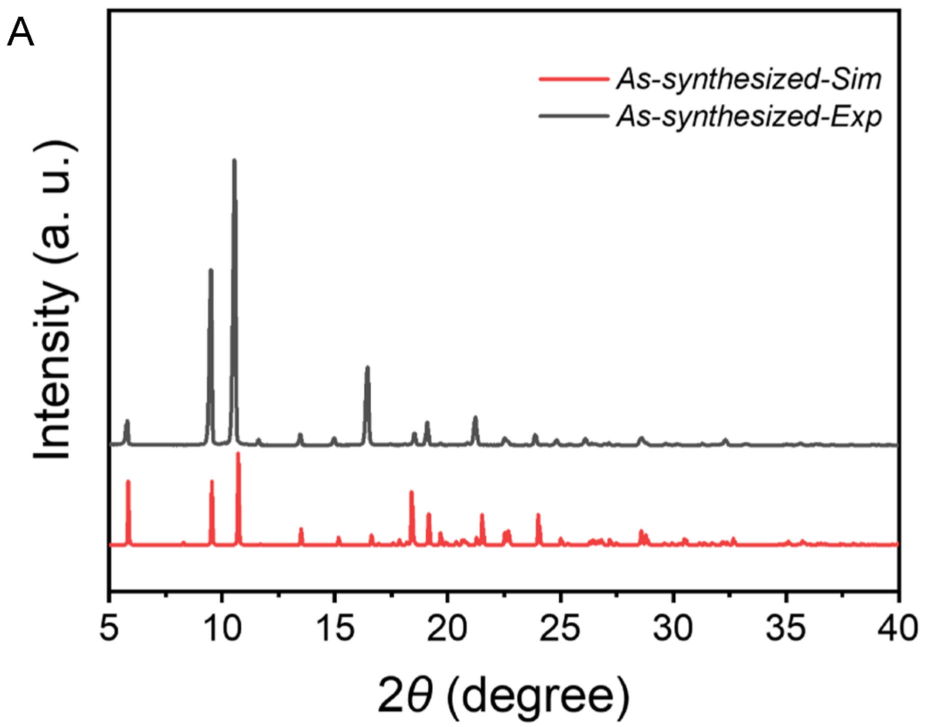

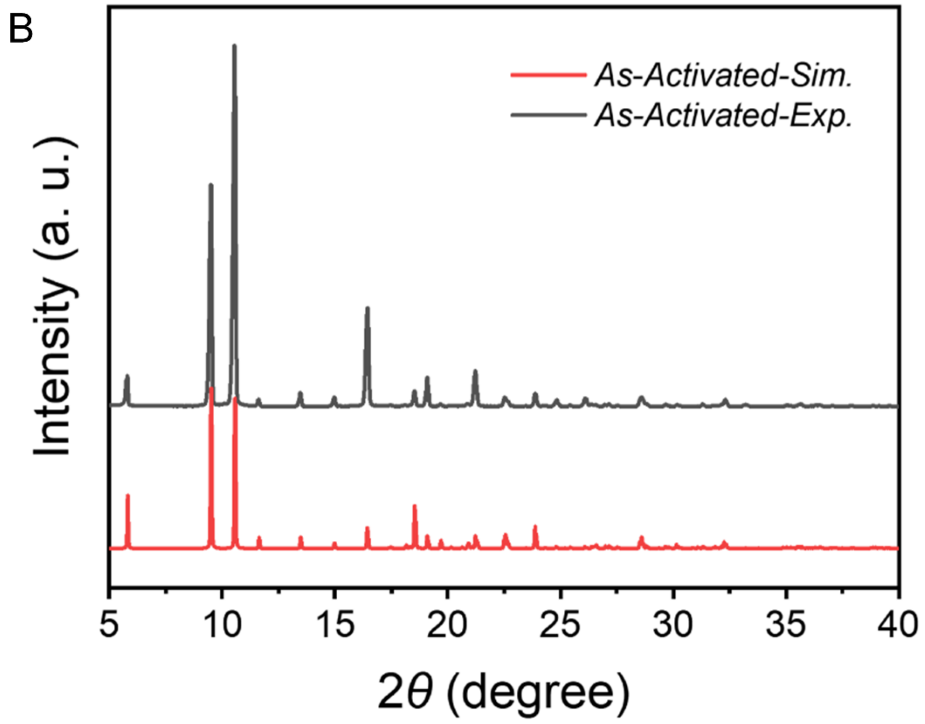

Fig. S5. The patterns of Powder X-ray diffraction (PXRD). (**A**) as-synthesized-Exp. (black) and as-synthesized-Sim. (red) of Ca-tcpb. (**B**) as-activated-Exp. (black) and as-activated-Sim. (red) of Ca-tcpb.

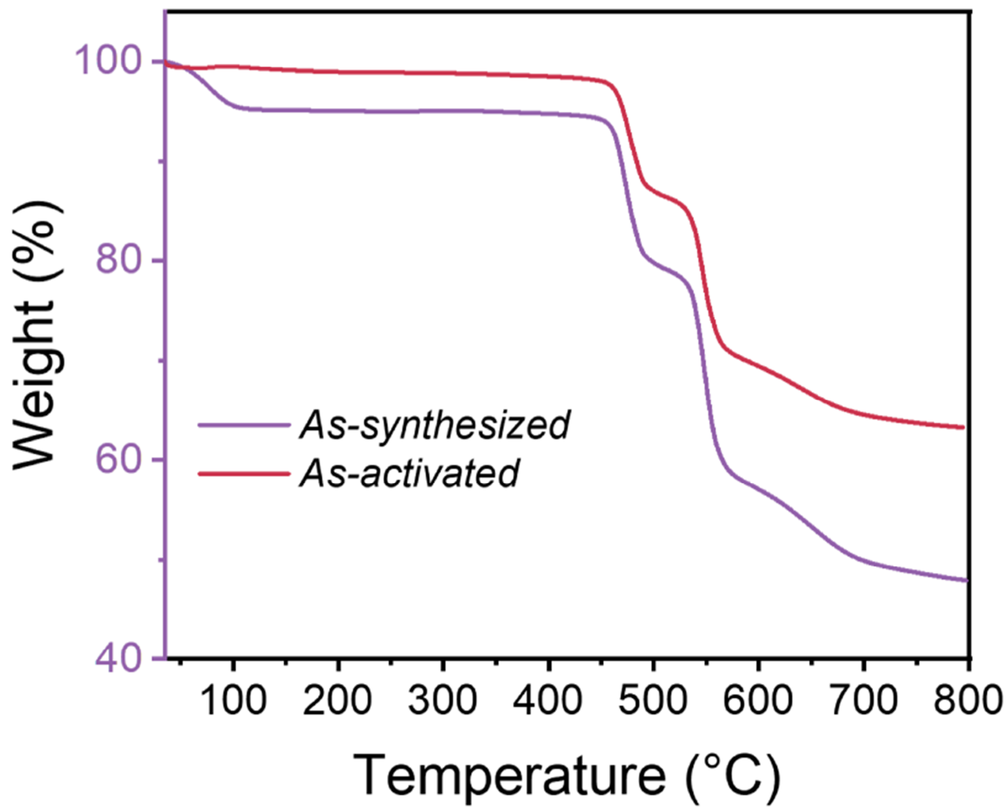

Fig. S6. Thermogravimetric analysis (TGA) curves of as-synthesized (purple) and as-activated (red) of Ca-tcpb.

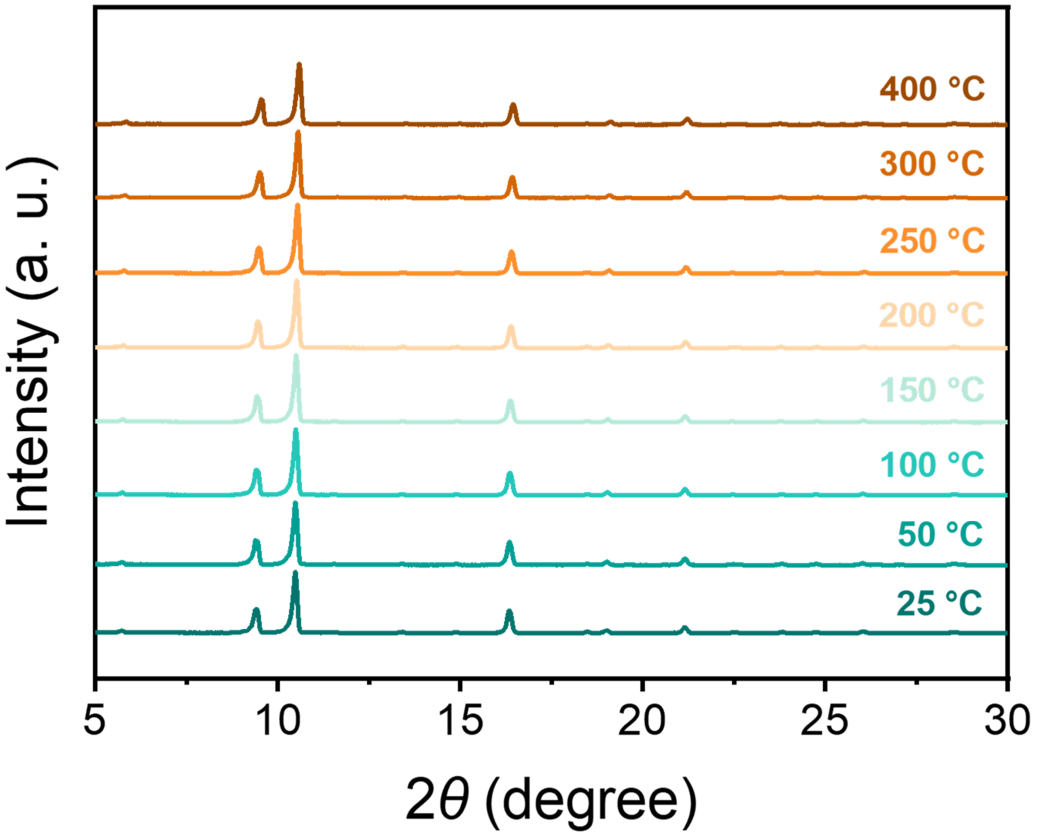

Fig. S7. Variable-temperature powder X-ray diffraction patterns of 1a.

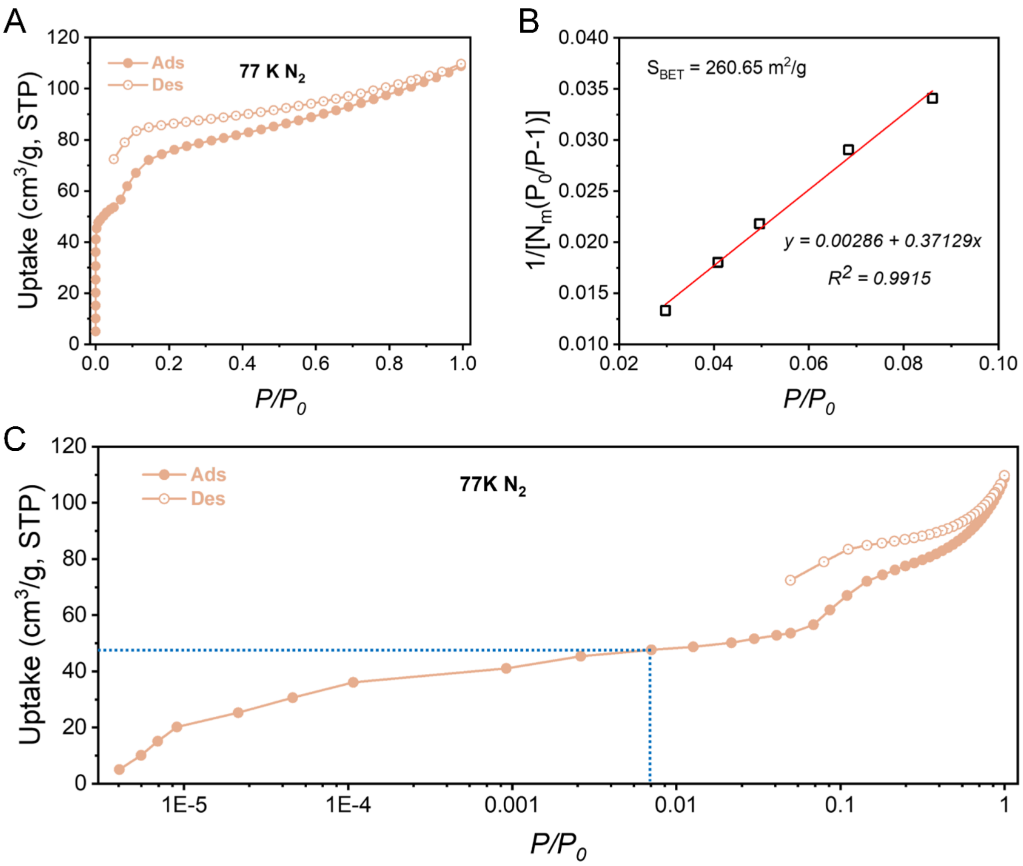

Fig. S8. N_2 sorption isotherms of 1a at 77 K. (**A**) and (**C**): Solid and open symbols represent adsorption and desorption branches, respectively. The x-axis is presented in logarithm in (**C**). (**B**) The calculated BET surface area of **1a**.

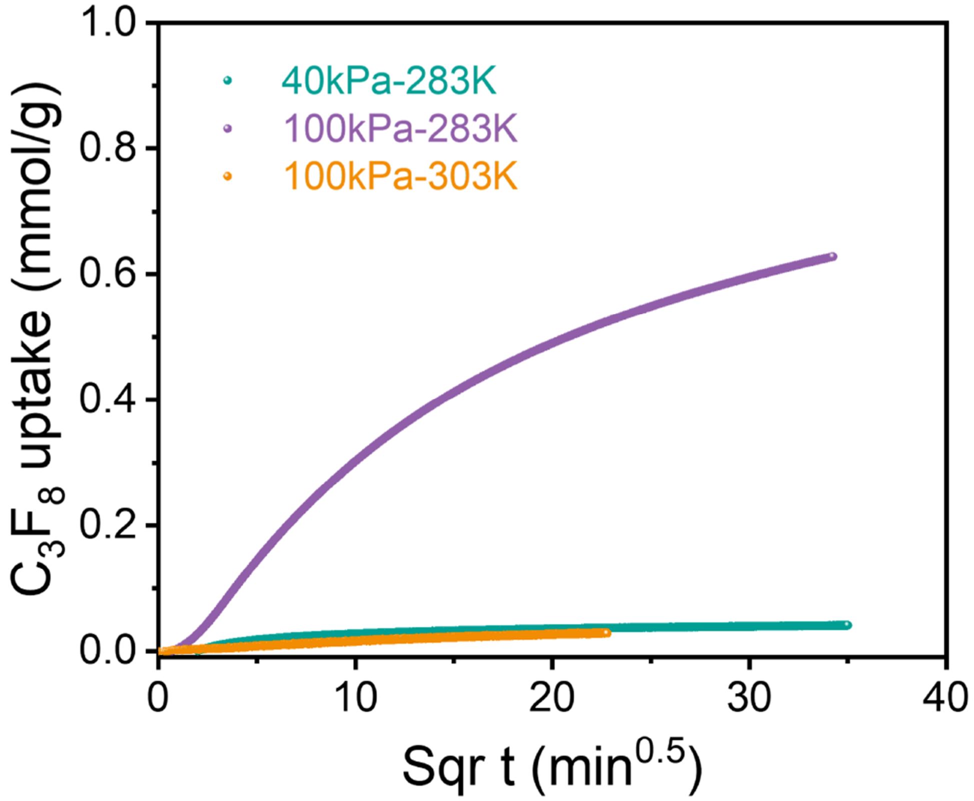

Fig. S9. C_3F_8 adsorption kinetic profiles for 1a at 283 and 303 K.

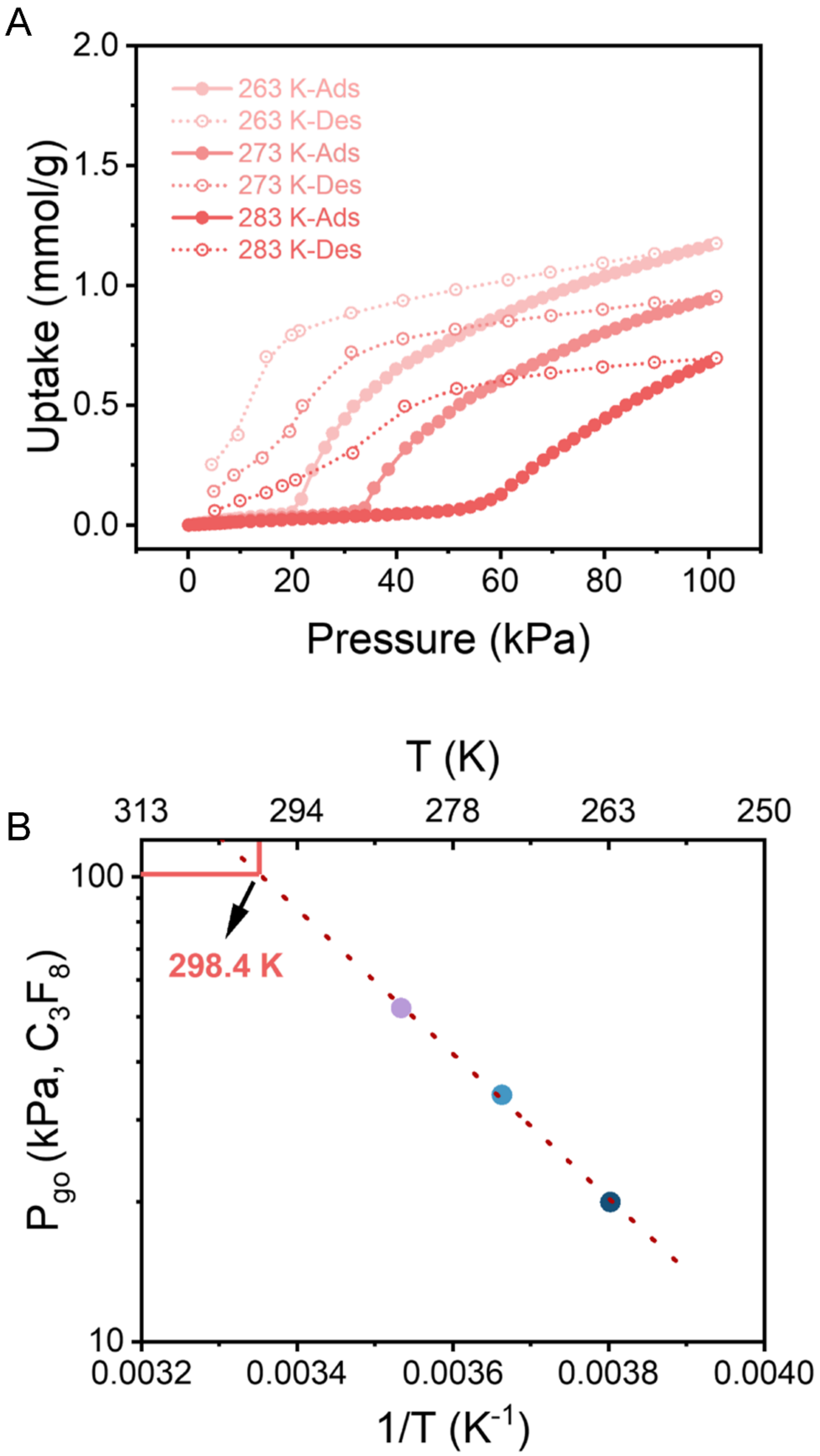

Fig. S10. Variable temperature C_3F_8 adsorption/desorption of 1a. (**A**) Solid (adsorption) and open (desorption) symbols. (**B**) Plot of gate opening pressure (P_{go}) vs inverse of measurement temperature (1/T) for adsorption isotherms of C_3F_8 measured at 263 (dark blue), 273 (light blue), 283 (purple) K. At P_{go} = 100 kPa (1 bar), the corresponding temperature (298.4 K) can be obtained (dotted lines).

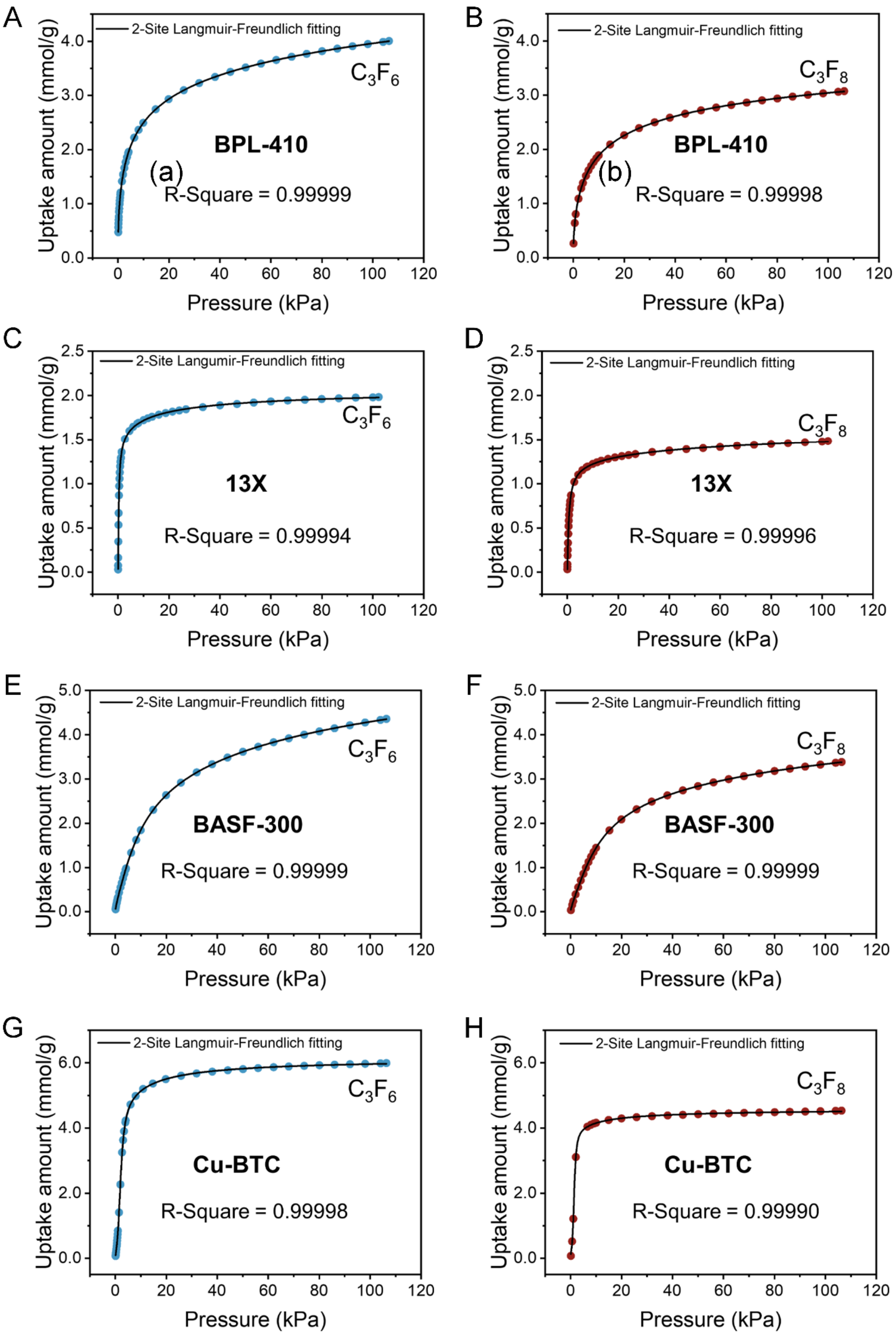

A
2-Site Langmuir-Freundlich fitting
C_3F_6
BPL-410
(a)
R-Square = 0.99999
B
2-Site Langmuir-Freundlich fitting
C_3F_8
BPL-410
(b)
R-Square = 0.99998
C
2-Site Langumir-Freundlich fitting
C_3F_6
13X
R-Square = 0.99994
D
2-Site Langmuir-Freundlich fitting
C_3F_8
13X
R-Square = 0.99996
E
2-Site Langmuir-Freundlich fitting
C_3F_6
BASF-300
R-Square = 0.99999
F
2-Site Langmuir-Freundlich fitting
C_3F_8
BASF-300
R-Square = 0.99999
G
2-Site Langmuir-Freundlich fitting
C_3F_6
Cu-BTC
R-Square = 0.99998
H
2-Site Langmuir-Freundlich fitting
C_3F_8
Cu-BTC
R-Square = 0.99990
Uptake amount (mmol/g)
Pressure (kPa)

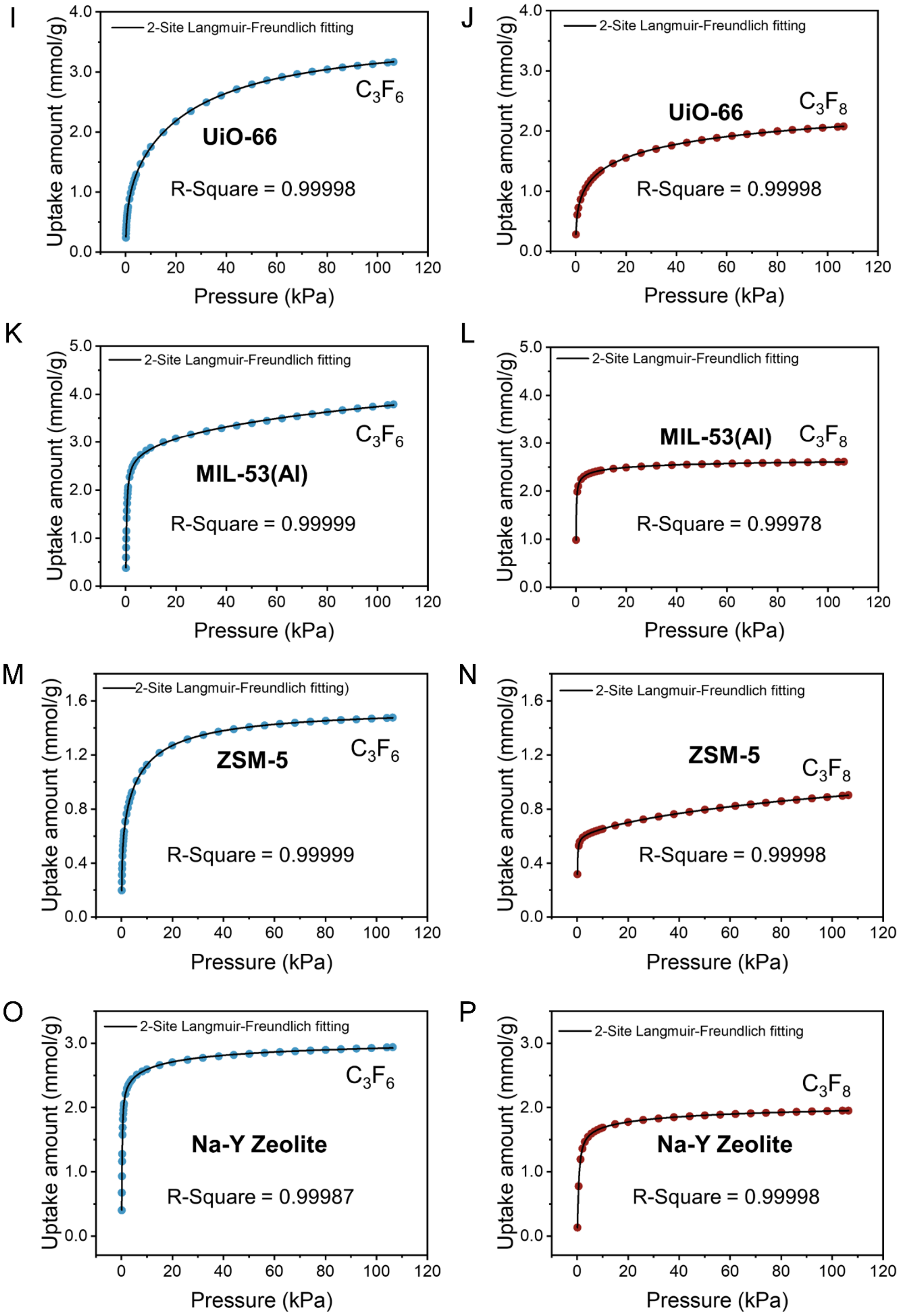
I
2-Site Langmuir-Freundlich fitting
C_3F_6
UiO-66
R-Square = 0.99998
Uptake amount (mmol/g)
Pressure (kPa)
J
2-Site Langmuir-Freundlich fitting
C_3F_8
UiO-66
R-Square = 0.99998
Uptake amount (mmol/g)
Pressure (kPa)
K
2-Site Langmuir-Freundlich fitting
C_3F_6
MIL-53(Al)
R-Square = 0.99999
Uptake amount (mmol/g)
Pressure (kPa)
L
2-Site Langmuir-Freundlich fitting
MIL-53(Al)
C_3F_8
R-Square = 0.99978
Uptake amount (mmol/g)
Pressure (kPa)
M
2-Site Langmuir-Freundlich fitting)
C_3F_6
ZSM-5
R-Square = 0.99999
Uptake amount (mmol/g)
Pressure (kPa)
N
2-Site Langmuir-Freundlich fitting
ZSM-5
C_3F_8
R-Square = 0.99998
Uptake amount (mmol/g)
Pressure (kPa)
O
2-Site Langmuir-Freundlich fitting
C_3F_6
Na-Y Zeolite
R-Square = 0.99987
Uptake amount (mmol/g)
Pressure (kPa)
P
2-Site Langmuir-Freundlich fitting
C_3F_8
Na-Y Zeolite
R-Square = 0.99998
Uptake amount (mmol/g)
Pressure (kPa)

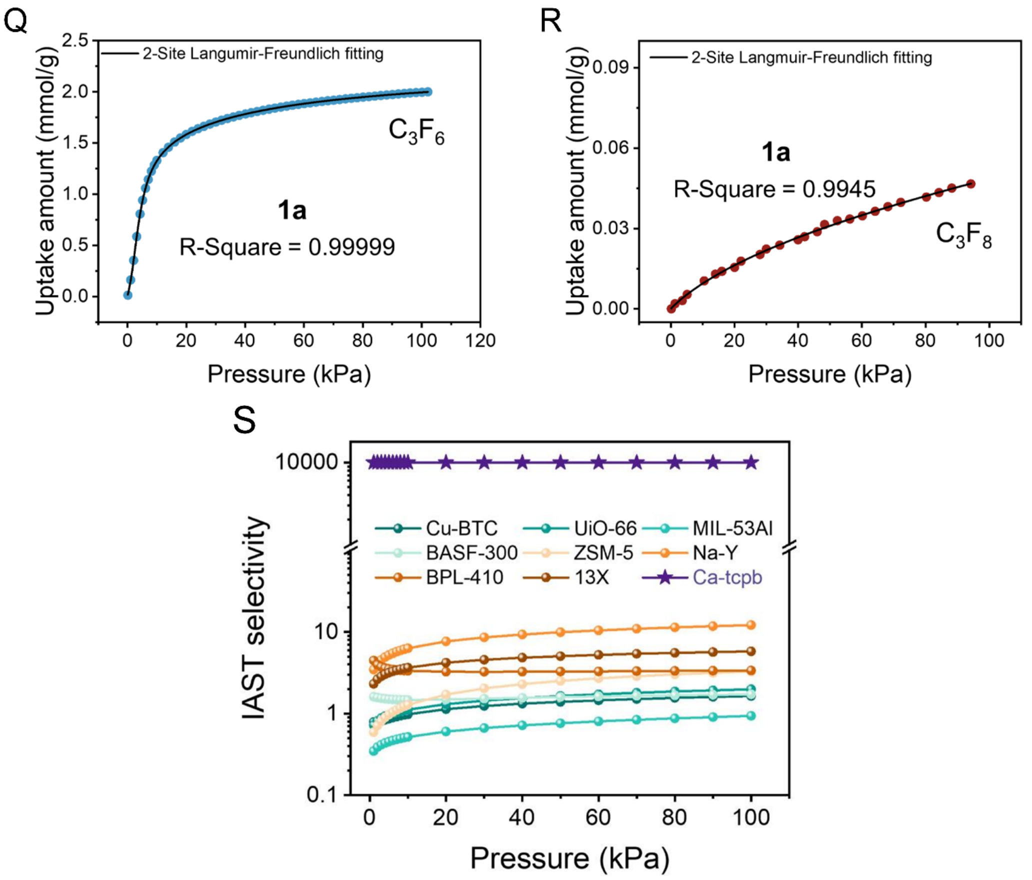

Fig. S11. C_3F_6 and C_3F_8 adsorption isotherms at 298 K in selected porous materials with dual-site Langmuir-Freundlich model fits. (**A**) and (**B**) BPL-410; (c) and (d) 13X; (e) and (f) BASF-300; (**G**) and (**H**) Cu-BTC; (**I**) and (**J**) UiO-66; (**K**) and (**L**) MIL-53 (Al); (**M**) and (**N**) ZSM-5; (**O**) and (**P**) Na-Y Zeolite; (**Q**) and (**R**) **1a**. (**S**) Comparison of C_3F_6/C_3F_8 (10/90) IAST selectivity of various porous solids.

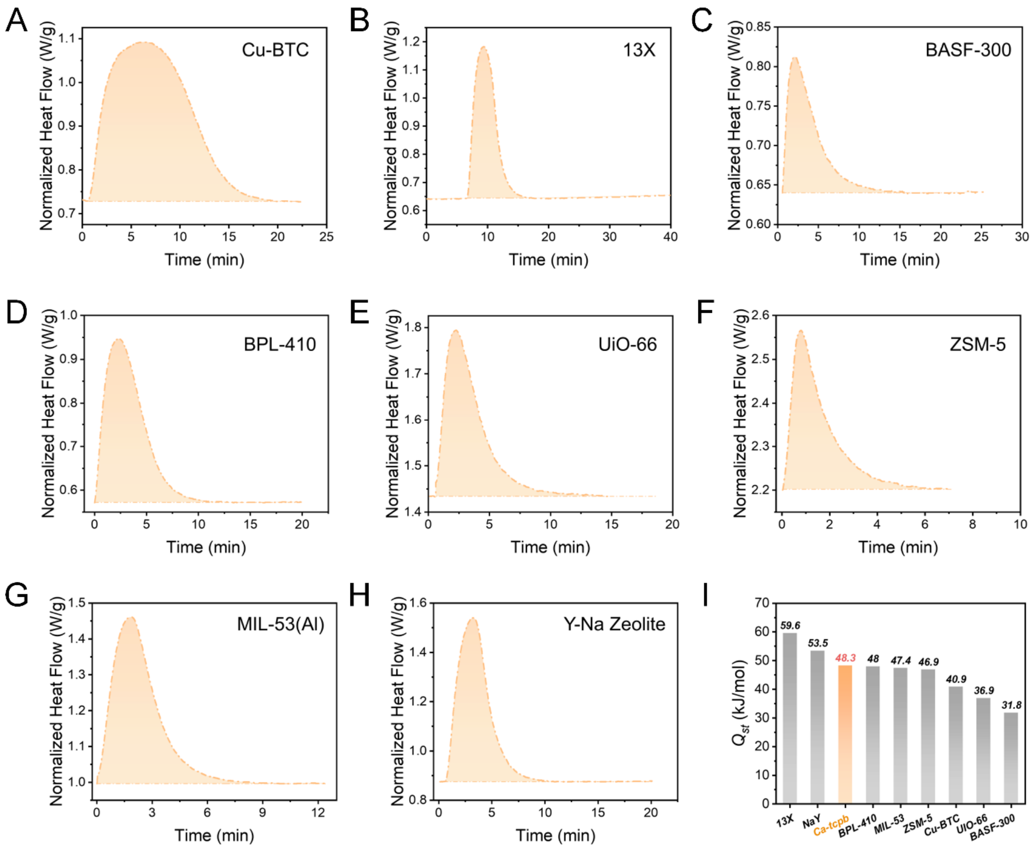

Fig. S12. The heat of adsorption for C_3F_6 on representative porous solids obtained by TG-DSC analysis. (**A**) Cu-BTC; (**B**) 13X; (**C**) BASF-300; (**D**) BPL-410; (**E**) UiO-66; (**F**) ZSM-5; (**G**) MIL-53(Al); (**H**) Y-Na Zeolite; (**I**) Comparison of Q_{st} values in these porous solids.

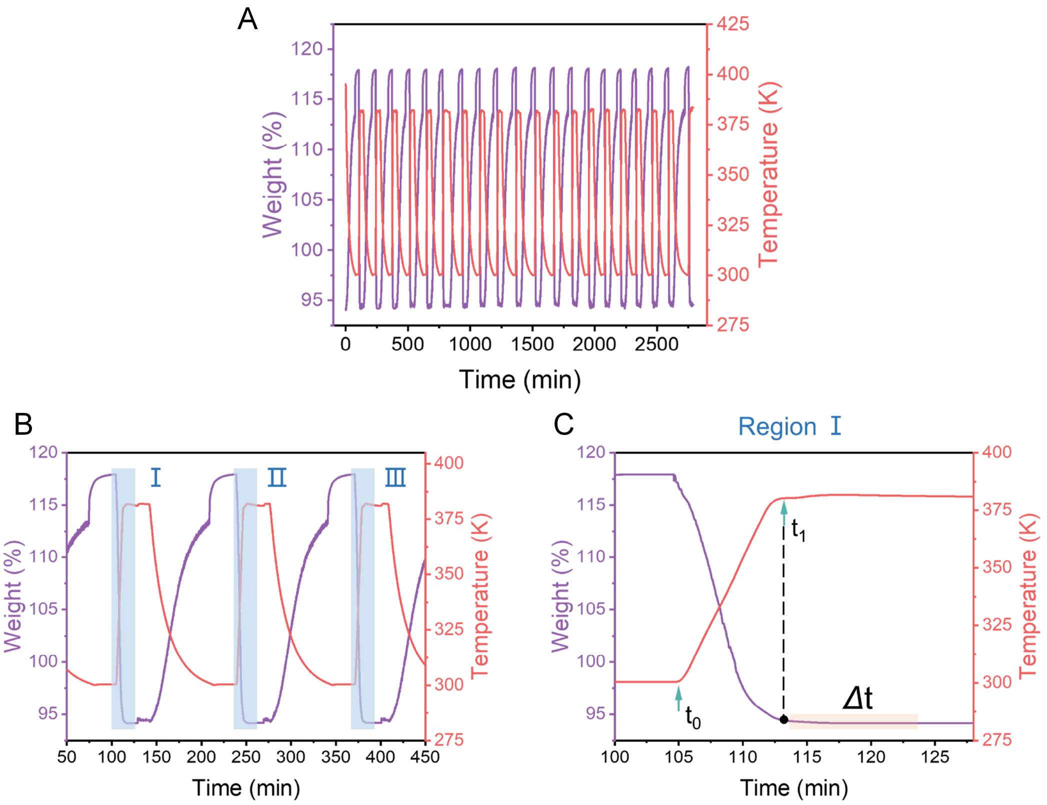

Fig. S13. TGA cycling studies of 1a. **(A)** C_3F_6 adsorption-desorption cycling test on **1a** for 20 consecutive adsorption cycles at 303 K. **(B)** and **(C)** Magnified views of **(A)**, showing the easy regeneration of **1a**.

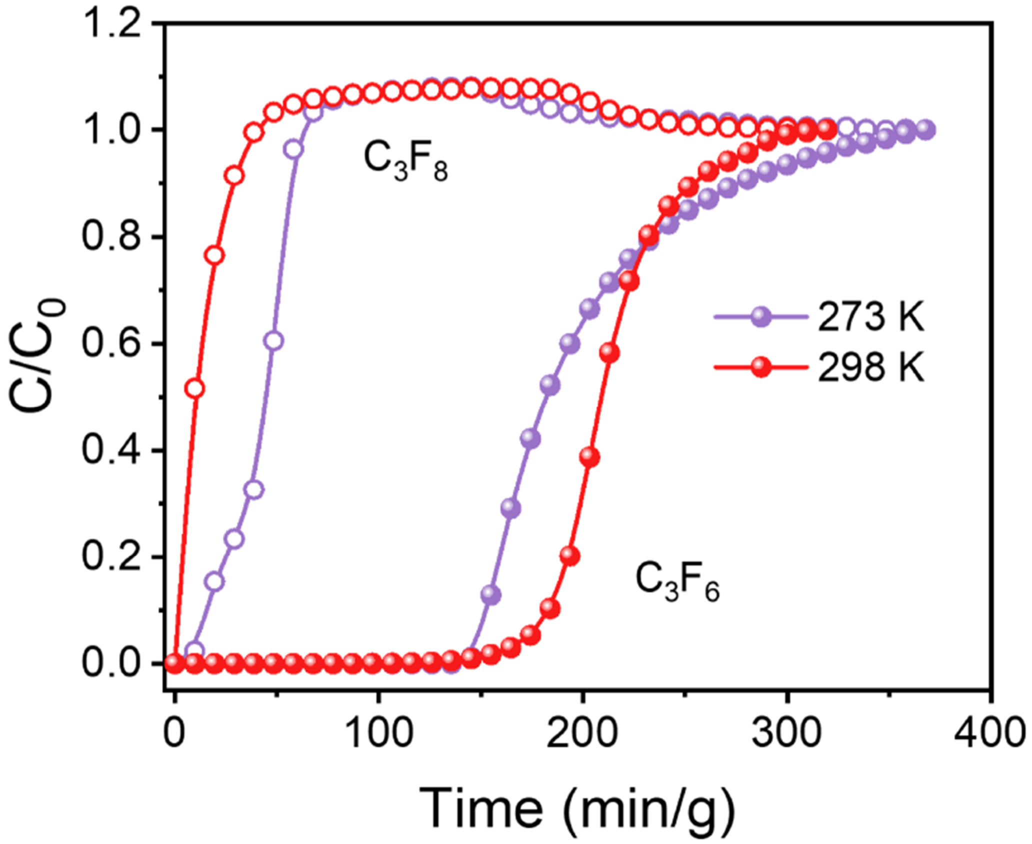

Fig. S14. Single dynamic breakthrough curves of a (10/90, v/v) C_3F_6/C_3F_8 gas mixtures using a packed column bed with 1a at 273, 283 K and a 1 bar.

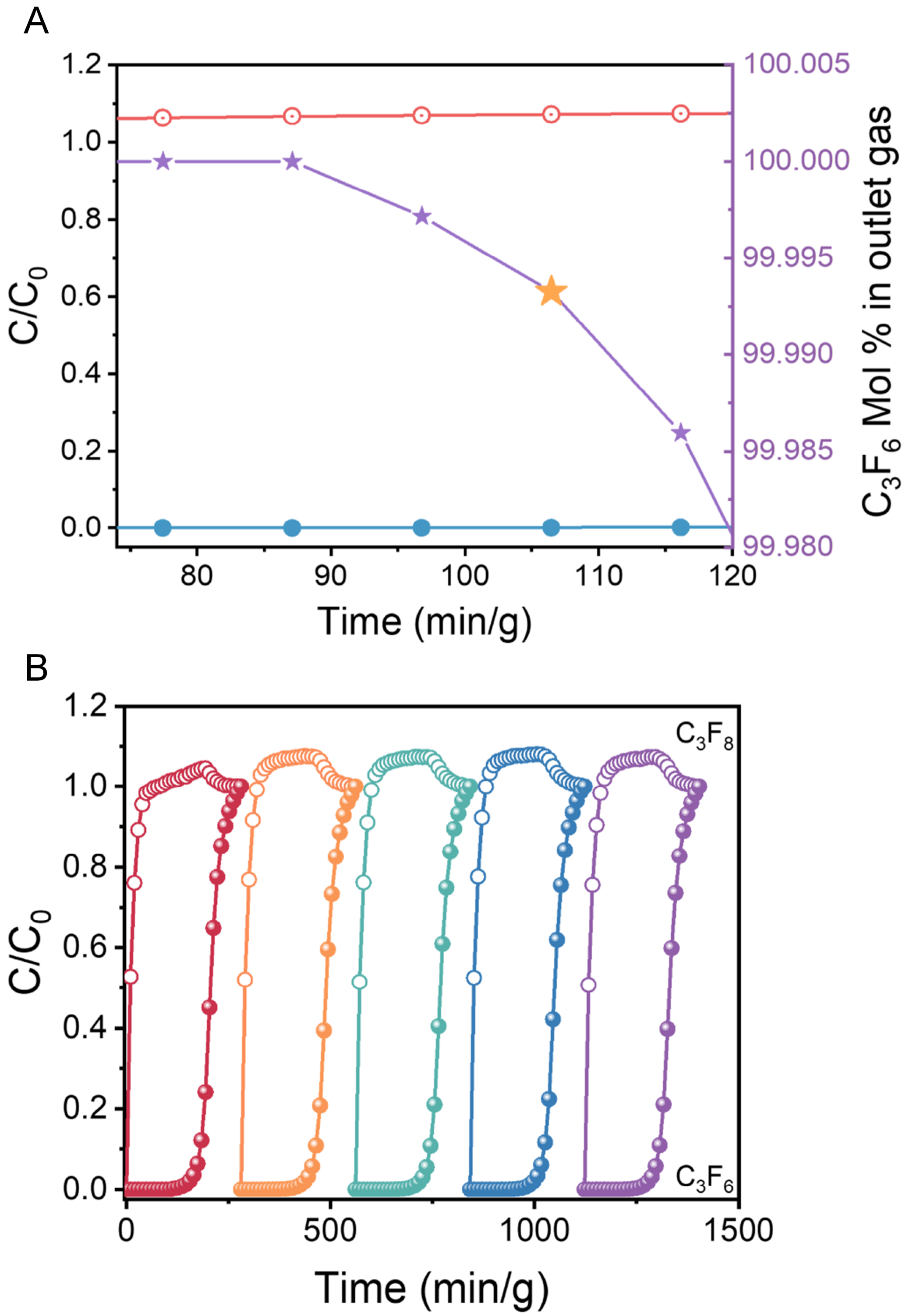

Fig. S15. Dynamic breakthrough studies of 1a. (**A**) Partially enlarged contour plot of **Fig. 2D**. (**B**) Multicycle dynamic breakthrough curves of a (10/90, v/v) C_3F_6/C_3F_8 gas mixtures using a packed column bed with **1a** at 298 K and 1 bar.

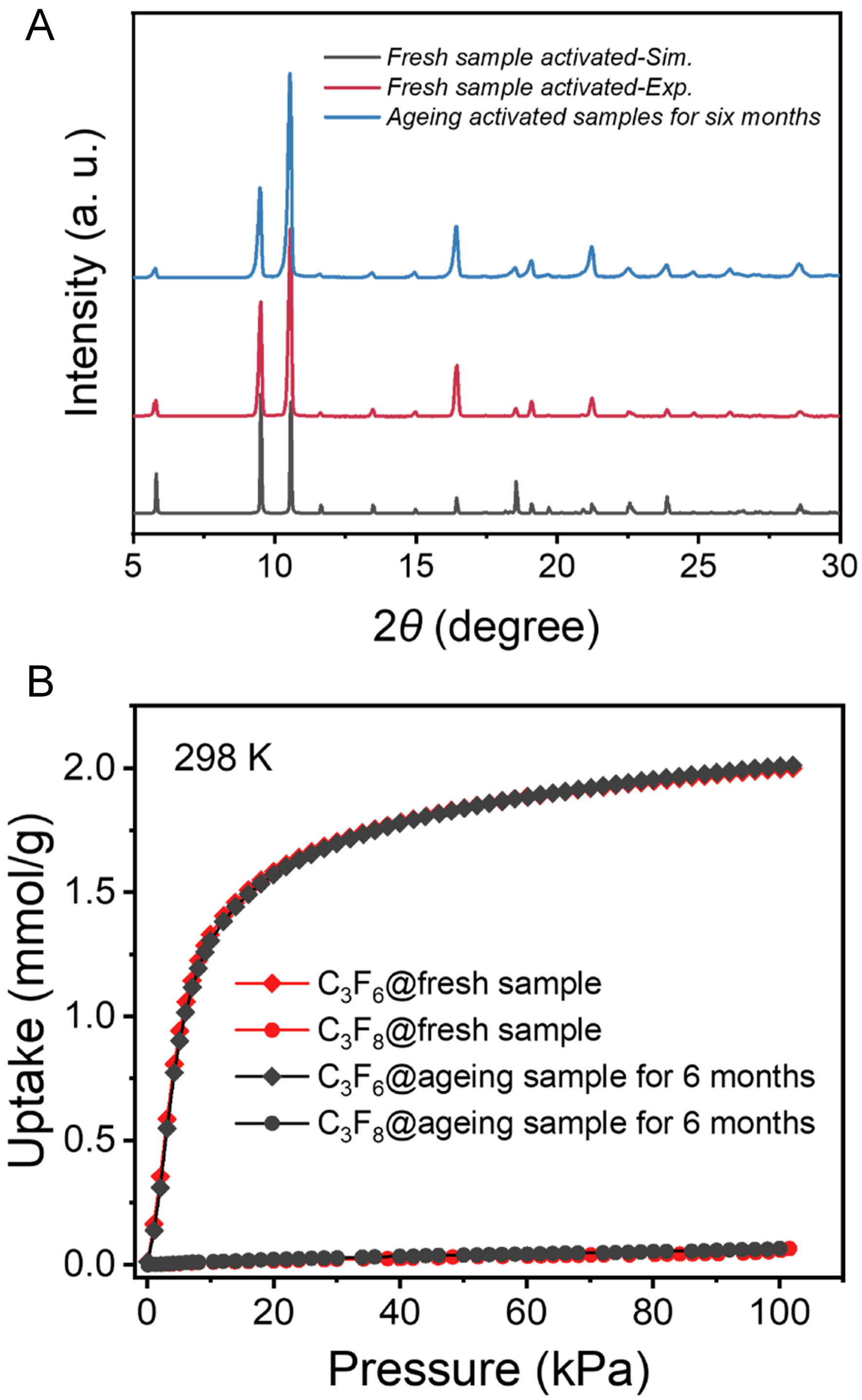

Fig. S16. Stability tests of 1a. (A) Powder X-ray diffraction (PXRD) patterns of samples fresh activated-Sim. (black), as-activated-Exp. (red) and ageing for six months (blue) of **1a**. **(B)** Single-component adsorption isotherms of C_3F_6 and C_3F_8 on **1a** before and after ageing.

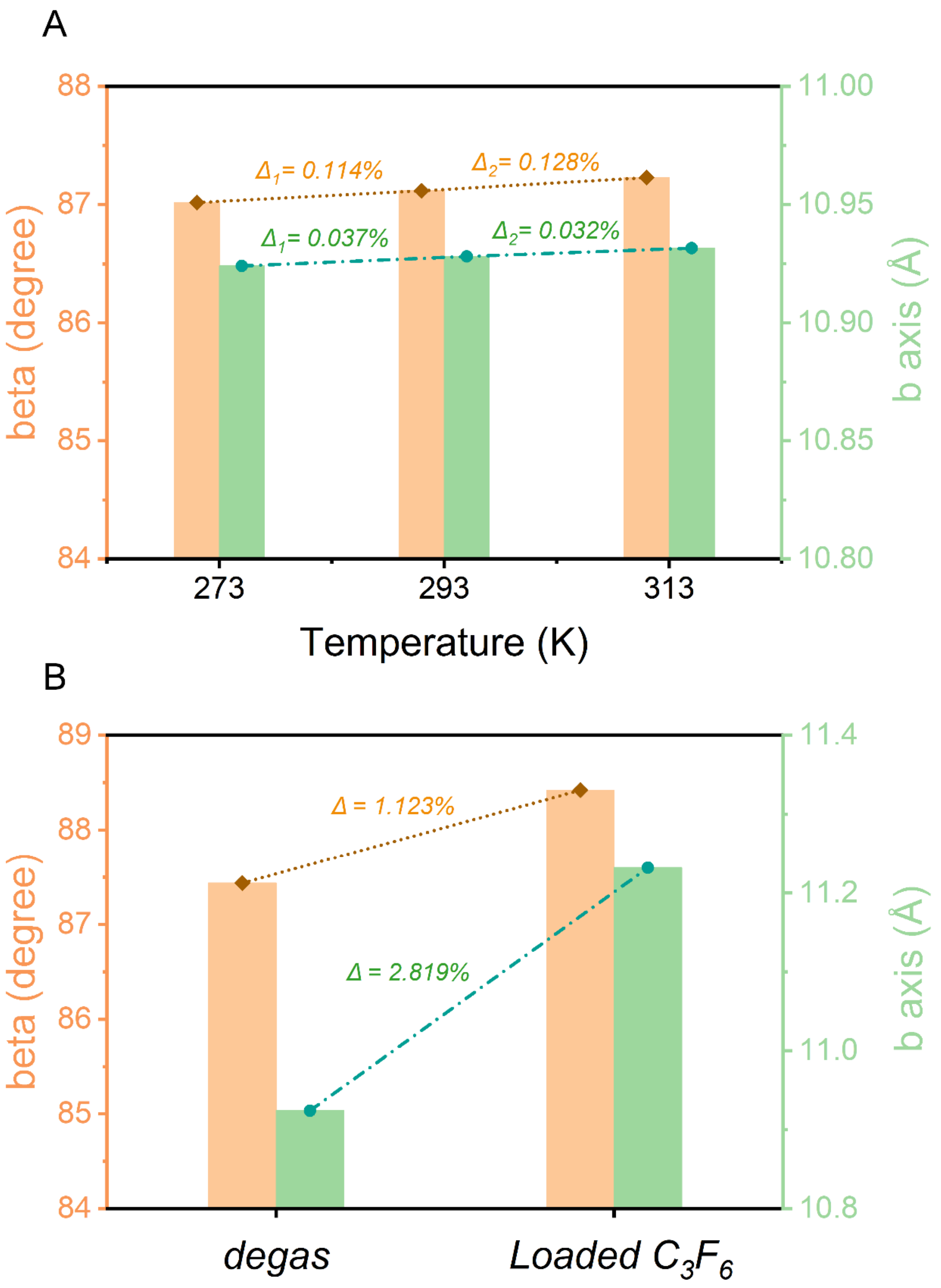

Fig. S17. The studies on the variation of cell parameters. (**A**) Cell length/angle of **1a** at different temperatures. The *b*-axes changes linearly by +0.03% and the *beta* angle changes by + 0.1% in the temperature range of 273-313 K; and (**B**) Comparison of cell parameters before and after loading C_3F_6. The *b*-axes and the *beta* angle show an increase of + 2.8%, + 1.1% on C_3F_6@**1a,** respectively.

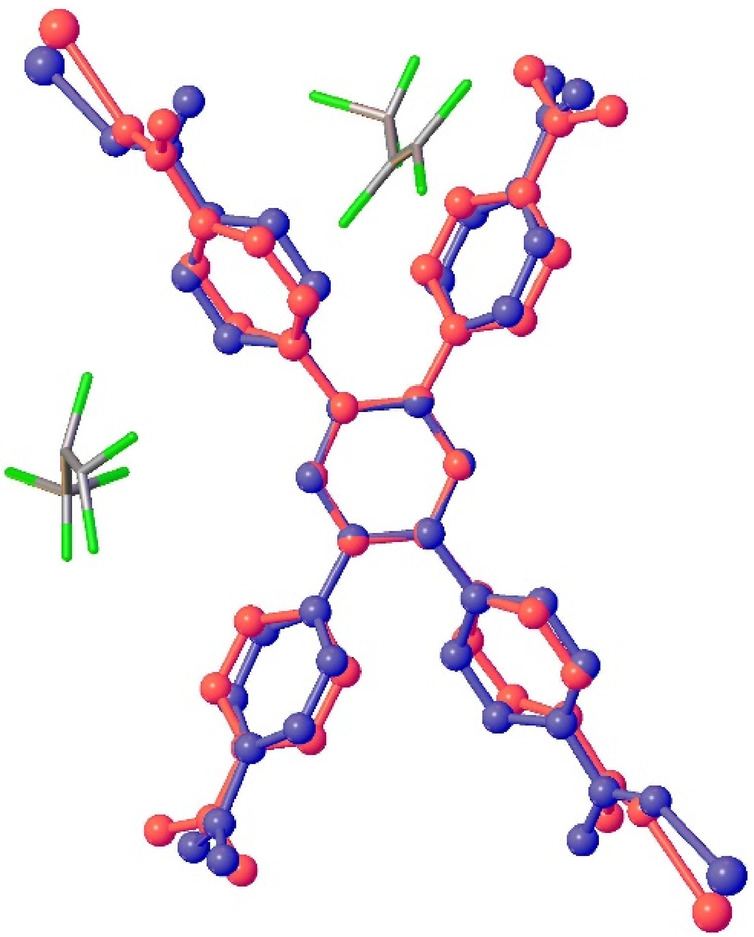

Fig. S18. Conformational comparisons of the $tcpb^{4-}$ linkers in the single-crystal structures of Ca-tcpb (red) and C_3F_6@Ca-tcpb (blue).

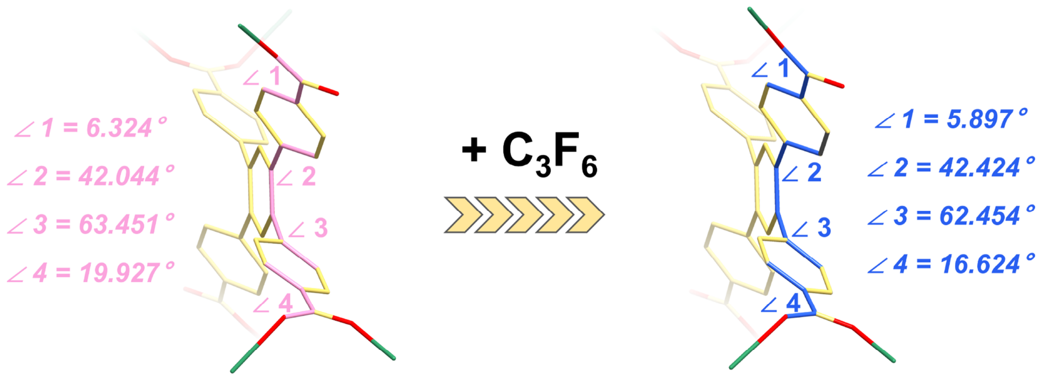

Fig. S19. The differences of ligand H_2tcpb^{2-} before and after adsorbing C_3F_6 of the framework. Dihedral angle (∠1, ∠2, ∠3, ∠4) representing the rotation angle of the single bond in carboxyphenyl.

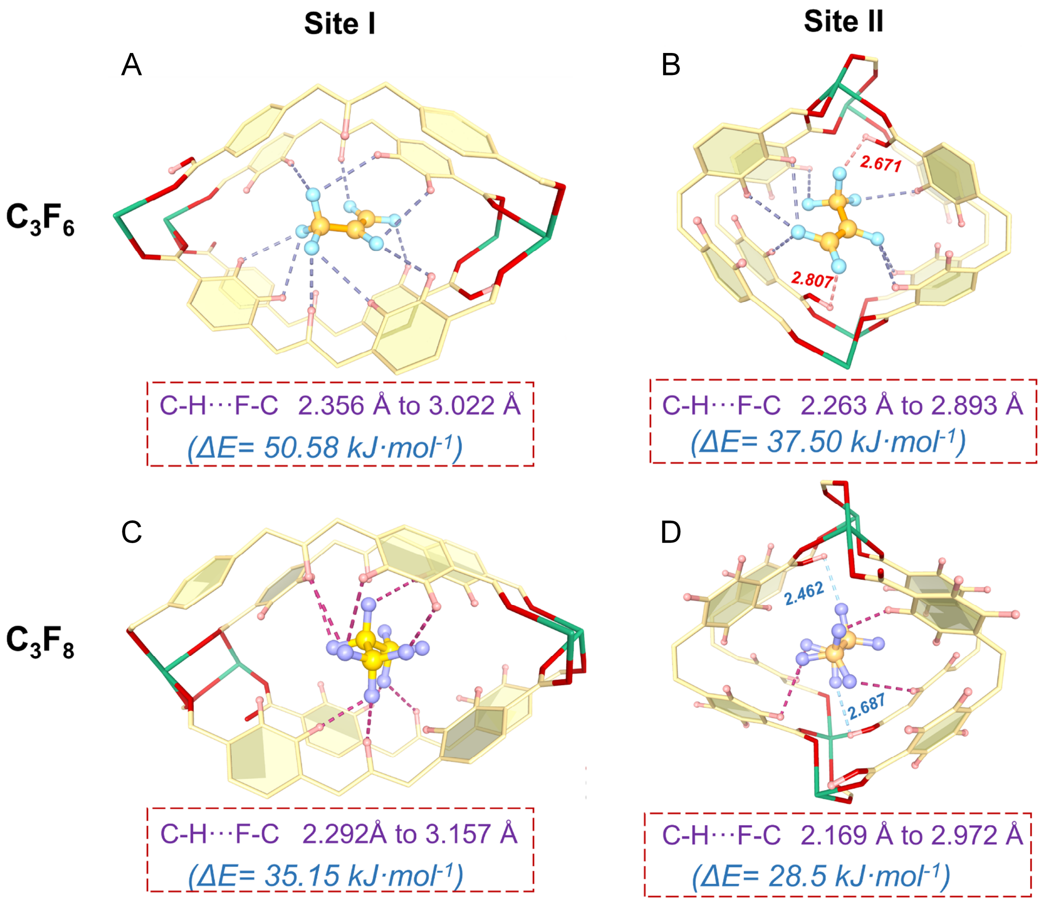

Fig. S20. The simulated adsorption sites: C_3F_6 (**A**) site I, (**B**) site II and C_3F_8 (**C**) site I, (**D**) site II in **1a**.

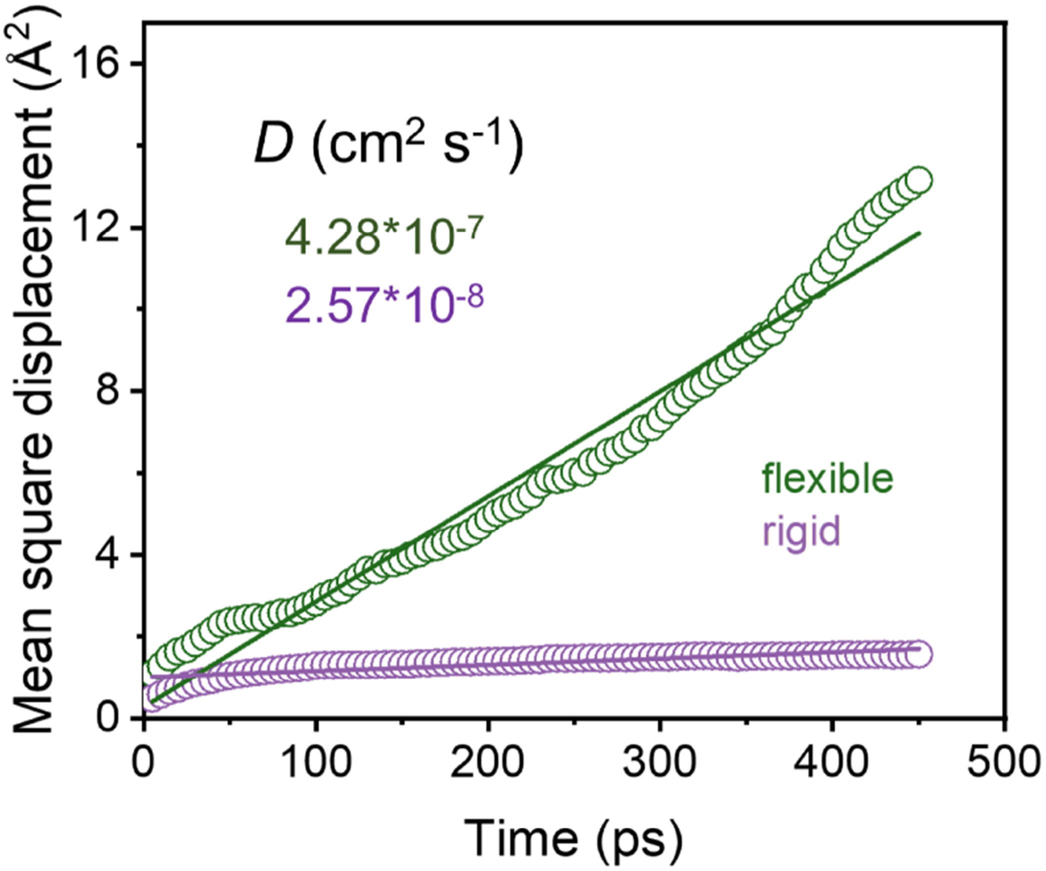

Fig. S21. MD simulated self-diffusion rates of C_3F_8 in 1a, considering the hosts are flexible and rigid.

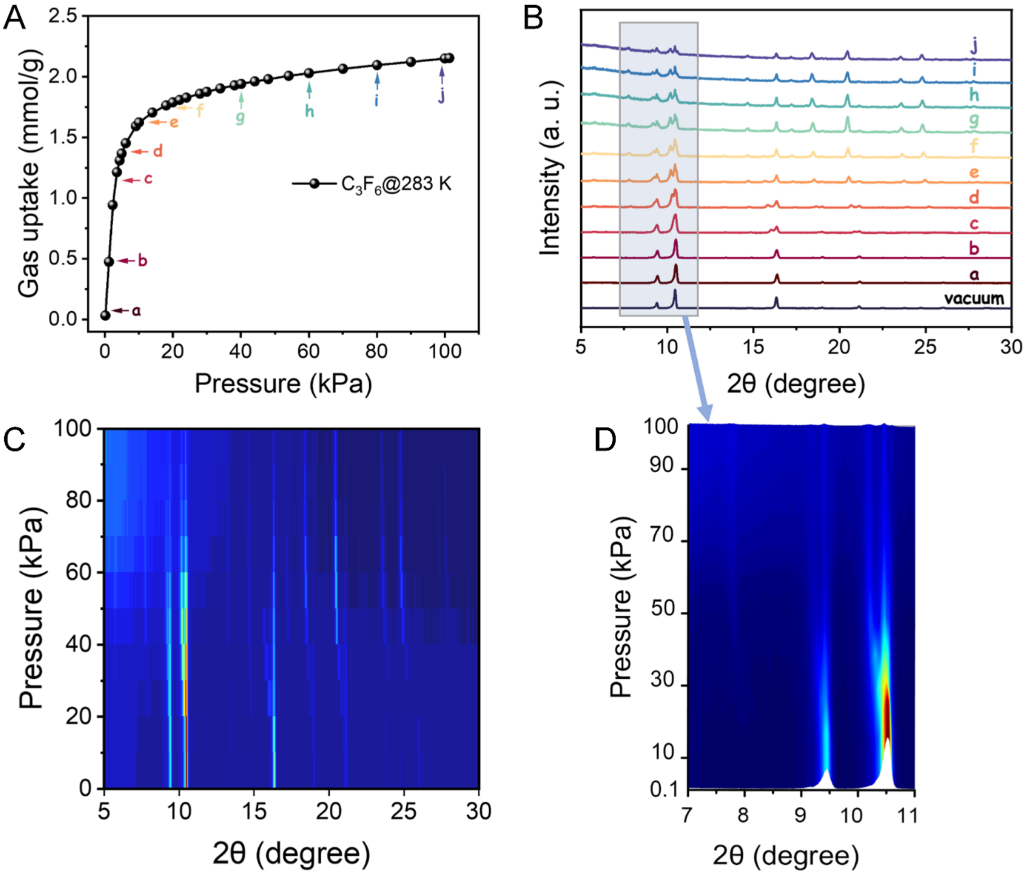
A
Gas uptake (mmol/g)
Pressure (kPa)
C3F6@283 K
a
b
c
d
e
f
g
h
i
j
B
Intensity (a. u.)
2θ (degree)
vacuum
C
Pressure (kPa)
2θ (degree)
D
Pressure (kPa)
2θ (degree)

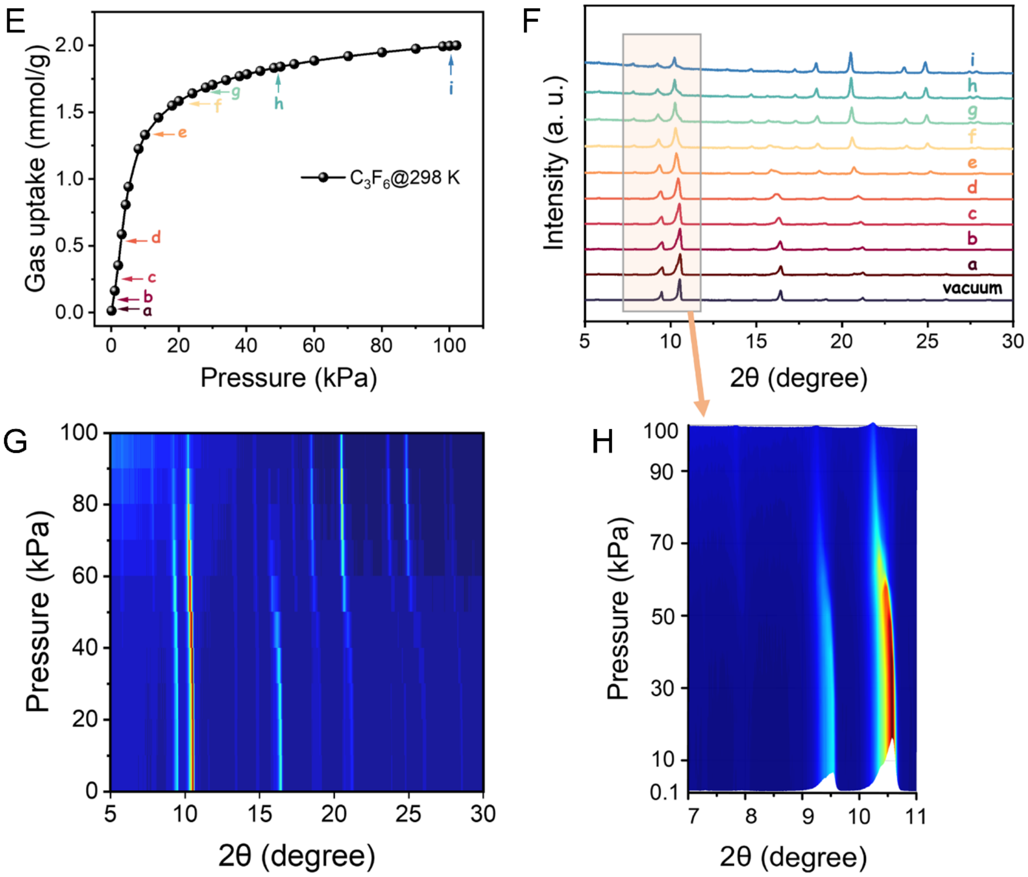

Fig. S22. *In-situ* C_3F_6-loaded PXRD. No. letters in adsorption correspond to the numbering of the PXRD patterns: (**A**) and (**E**) Adsorption isotherm at 283 K and 298 K. (**B**) and (**F**) PXRD pattern recorded during loading C_3F_6 under different pressures at 283 K and 298 K. (c) and (g) of the PXRD pattern evolution of C_3F_6 at 283 K and 298 K. (**D**) and (**H**) Partially enlarged contour plot.

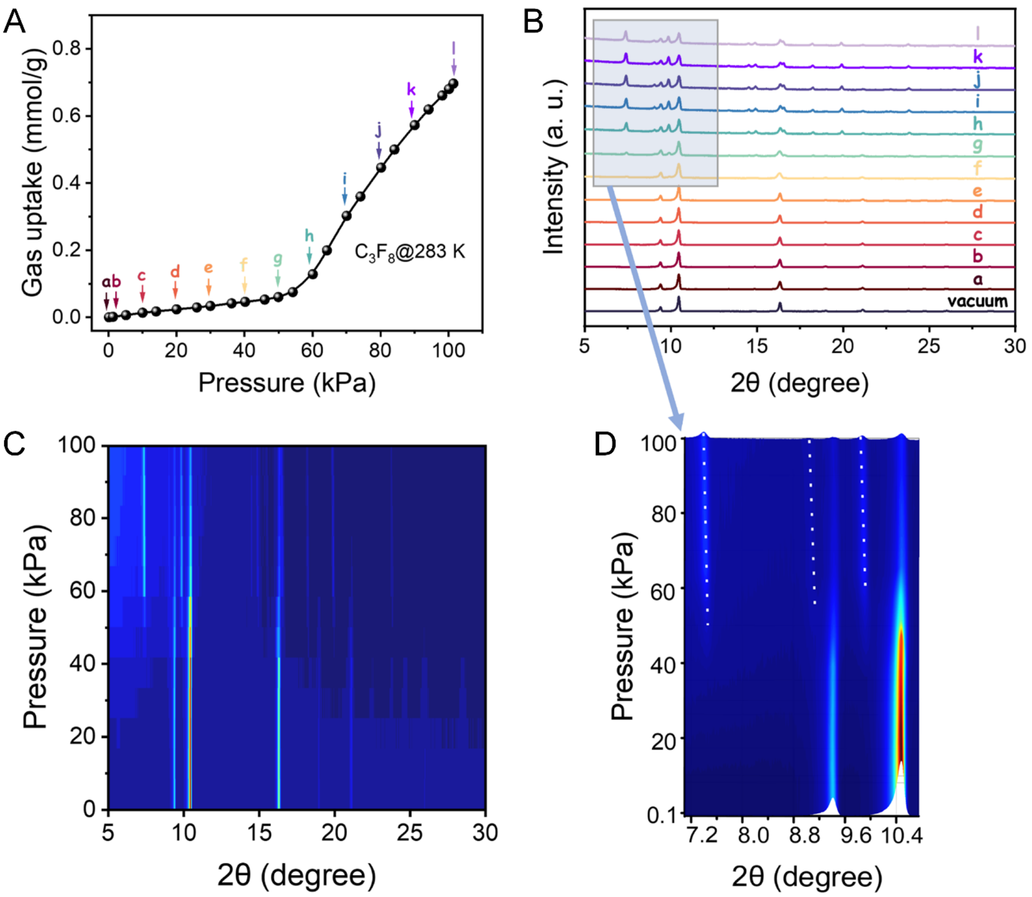

A
Gas uptake (mmol/g)
Pressure (kPa)
C3F8@283 K
a b c d e f g h i j k l
B
Intensity (a. u.)
2θ (degree)
vacuum
C
Pressure (kPa)
2θ (degree)
D
Pressure (kPa)
2θ (degree)

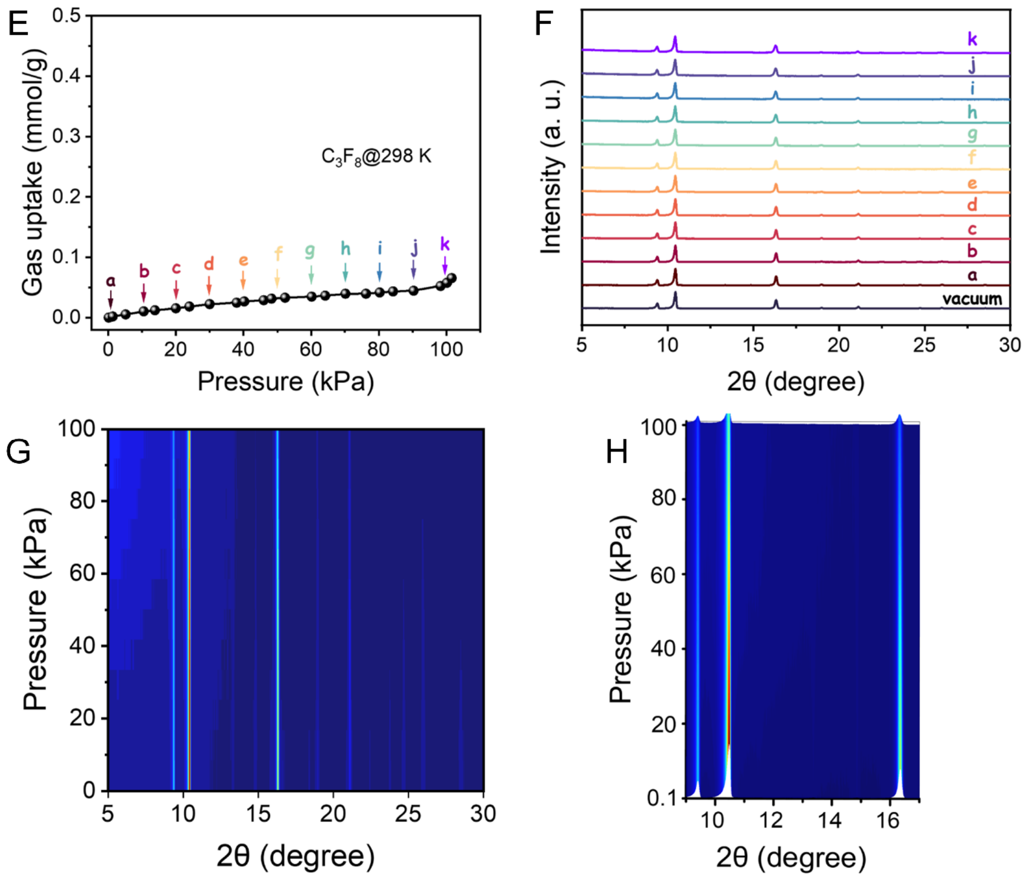

Fig. S23. ***In-situ*** **C_3F_8-loaded PXRD.** No. letters in adsorption correspond to the numbering of the PXRD patterns: (**A**) and (**E**) Adsorption isotherm at 283 K and 298 K. (**B**) and (**F**) PXRD pattern recorded during loading C_3F_8 under different pressures at 283 K and 298 K. (c) and (g) of the PXRD pattern evolution of C_3F_8 at 283 K and 298 K. (**D**) and (**H**) Partially enlarged contour plot.

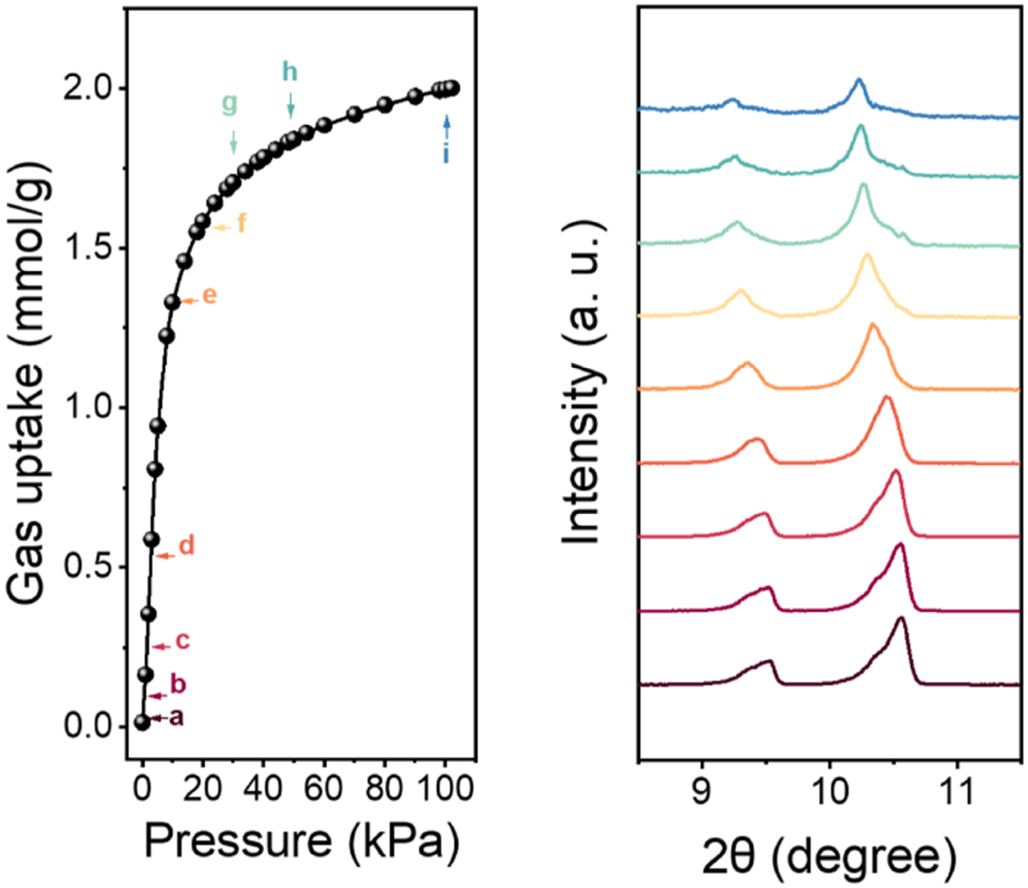

Fig. S24. Adsorption isotherms (left) and in situ adsorption-XRD (right) of 1a for C_3F_6 at 298 K.

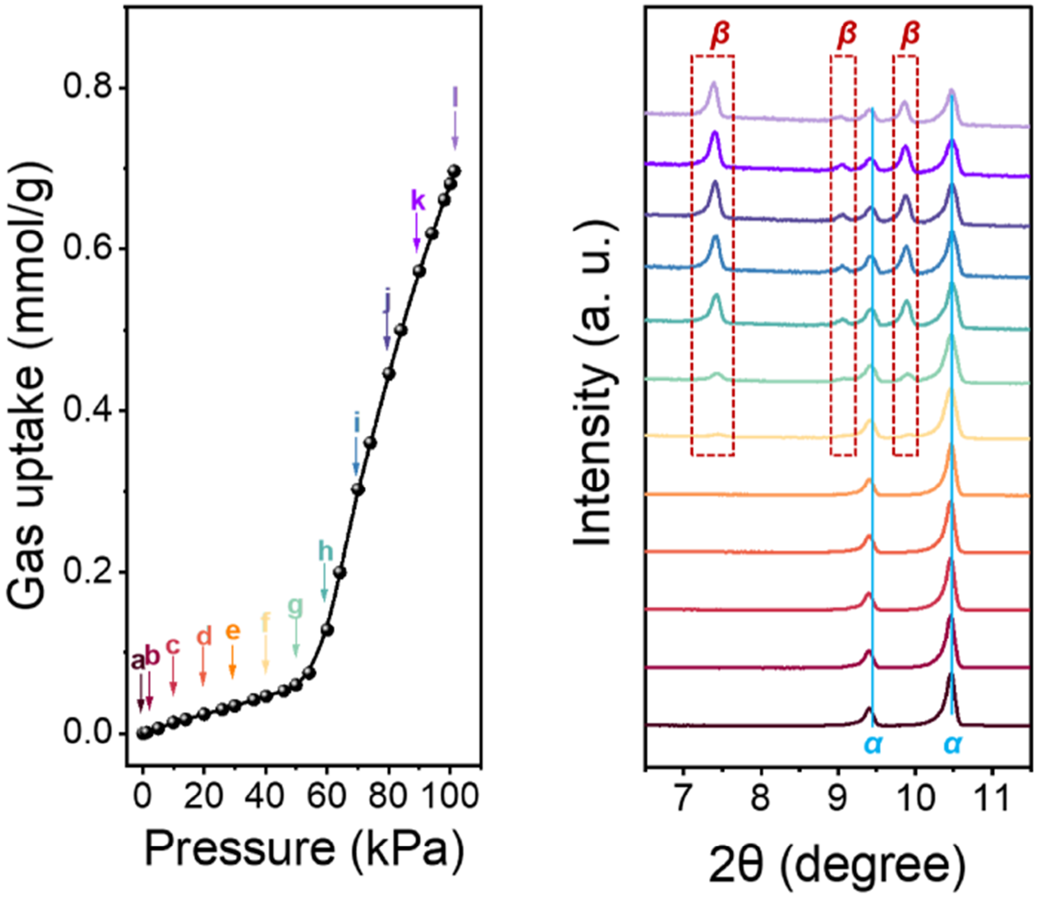

Fig. S25. Adsorption isotherms (left) and in situ adsorption-XRD (right) of 1a for C_3F_8 at 283 K.

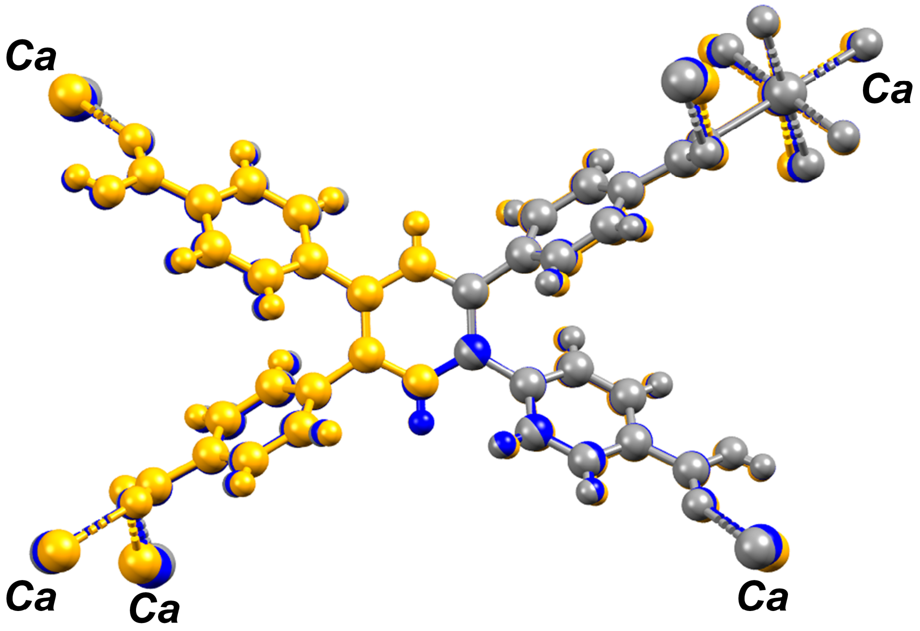

Fig. S26. The overlay diagram of the variable temperature single crystals of 1a.

Table S1. Reference to synthesis or vendor of the materials.

Material	Synthesis	Purchase [a]	Activation conditions (Dynamic vacuum)
13X	-	Sigma Aldrich	24h, 200°C
Basolite F300	-	Sigma Aldrich	24h, 120°C
BPL-410	-	Calgon Carbon	24h, 200°C
Zeolite Y (NaY)	-	XFNANO	24h, 200°C
ZSM-5	-	XFNANO	24h, 200°C
UiO-66	Bárcia *et al* (*64*).	-	24h, 150°C
Cu-BTC	Li *et al* (*65*).	-	24h, 120°C
MIL-53(Al)	Wang *et al* (*66*).	-	24h, 120°C
Ca-tcpb	Li *et al (46).*		24h, 150°C

[a] Used without further purification

Table S2. The fitted parameters for the dual-site Langmuir-Freundlich equation for the single component isotherms of C_3F_6 in measured porous solids at 298 K.

Adsorbents	$q_{A,sat}$	b_A	n_A	$q_{B,sat}$	b_B	n_B
BPL-410	2.321786	0.391295	0.620655	13.95717	0.041695	0.293398
13X	0.816169	0.250463	0.661889	1.290034	5.651764	1.431551
BASF-300	5.361961	0.051866	0.666812	1.501727	0.034555	1.529248
Cu-BTC	2.853365354	0.246913	0.879146	3.297751	0.083111	3.242826
UiO-66	1.846076	0.023704	1.102606	1.854056	0.62726	0.595797
MIL-53 (Al)	6.959922	0.040927	0.423858	2.185635	4.669427	1.46901
ZSM-5	0.98041	0.171108	0.892546	0.575586	5.361989	1.069896
NaY zeolite	1.286407	0.378544	0.547621	1.860418	12.48072	1.806859
**Ca-tcpb**	0.997588	0.045988	2.230768	1.283211	0.10082	0.784661

Table S3. The fitted parameters for the dual-site Langmuir-Freundlich equation for the single component isotherms of C_3F_8 in measured porous solids at 298 K.

**Adsorbents**	$q_{A,sat}$	b_A	n_A	$q_{B,sat}$	b_B	n_B
BPL-410	10.48623	0.018108	0.307054	2.781615	0.28849	0.617574
13X	0.78499	0.120875	0.520384	1.029343	1.910106	1.208212
BASF-300	1.392999	0.028803	1.578895	3.901208	0.049935	0.657356
Cu-BTC	3.023611	0.241316	3.849429	1.597927	0.46608	0.713688
UiO-66	0.055277	315.897	5.321528	3.06451	0.272471	0.42077
MIL-53 (Al)	1.508878	23.0834	1.535145	1.264425	1.130783	0.376896
ZSM-5	0.8709	0.015221	0.777019	0.585475	13.30359	1.037291
NaY zeolite	1.344601	2.066983	1.471569	0.822547	0.226549	0.540419
**Ca-tcpb**	1.605771	1.98E-18	8.058966	0.098444	0.036632	0.8323441

Table S4. Crystallographic data and structural refinement summary.

Compounds	**1a** under varied temperature				C_3F_6@**1a**
CCDC	2277051	2277052	2277053	2277054	2277055
Empirical formula	$C_{34}H_{20}CaO_8$	$C_{34}H_{20}CaO_8$	$C_{34}H_{20}CaO_8$	$C_{34}H_{20}CaO_8$	$C_{40}H_{20}CaF_{12}O_8$
Formula weight	596.58	596.58	596.58	596.58	896.64
Temperature/K	170.0	273.0	293.0	313.0	170.0
Crystal system	triclinic	triclinic	triclinic	triclinic	triclinic
Space group	*P-1*	*P-1*	*P-1*	*P-1*	*P-1*
a/Å	5.1186(2)	5.1340(2)	5.1369(2)	5.1390(2)	5.1039(11)
b/Å	10.8851(5)	10.9241(4)	10.9281(4)	10.9316(4)	11.232(3)
c/Å	15.2873(6)	15.2987(6)	15.3022(6)	15.3097(6)	15.317(3)
α/°	83.256(4)	83.317(2)	83.331(2)	83.354(2)	83.676(7)
β/°	86.531(3)	87.018(2)	87.1170(10)	87.229(2)	88.420(7)
γ/°	82.958(3)	82.880(2)	82.851(2)	82.831(2)	83.908(7)
Volume/$Å^3$	838.56(6)	845.00(6)	846.01(6)	847.10(6)	867.7(3)
Z	2	1	1	1	1
D_c (g/cm^3)	1.177	1.172	1.171	1.169	1.716
μ/mm^{-1}	2.003	0.231	0.231	0.231	0.304
F(000)	306.0	308.0	308.0	308.0	452.0
Crystal size/mm^3	0.1 × 0.03 × 0.02	0.45 × 0.05 × 0.04	0.45 × 0.05 × 0.04	0.45 × 0.05 × 0.04	0.1 × 0.03 × 0.02
Radiation	Cu Kα (λ = 1.54184)	Mo Kα (λ = 0.71073)	Mo Kα (λ = 0.71073)	Mo Kα (λ = 0.71073)	Mo Kα (λ = 0.71073)
Goodness-of-fit on F^2	1.120	1.035	1.039	1.037	1.039
Final R indexes [I>=2σ (I)] [(a)]	R_1 = 0.0383, wR_2 = 0.1126	R_1 = 0.0375, wR_2 = 0.0889	R_1 = 0.0379, wR_2 = 0.0904	R_1 = 0.0392, wR_2 = 0.0897	R_1 = 0.0747, wR_2 = 0.1896
Final R indexes [all data] [(a)]	R_1 = 0.0413, wR_2 = 0.1148	R_1 = 0.0507, wR_2 = 0.0972	R_1 = 0.0520, wR_2 = 0.0997	R_1 = 0.0532, wR_2 = 0.0983	R_1 = 0.1096, wR_2 = 0.2159
Largest diff. peak/hole / e$Å^{-3}$	0.58/-0.23	0.28/-0.20	0.23/-0.19	0.27/-0.21	0.80/-0.56

(a) $R_1=\sum\left||F_0|-|F_c|\right|/\sum|F_0|$;$wR_2=\left[\sum w(|F_0|^2-|F_c|^2)^2/\sum w(F_0^2)^2\right]^{1/2}$

Table S5. The price of large-scale production of MOF.

Ingredients	Unit price, USD, $/kg	Yield	Usage, kg	Total price, USD, $
Calcium chloride	7.23		2.20-2.93	15.9-21.2
1,2,4,5-Tetrakis(4-carboxyphenyl) benzene, H_4TCPB	4393.6	35-45 %, (based on Ca^{2+})	2.21-2.98	9709.8-13092.9
Ethanol absolute	0.9		190-200	171-180

REFERENCES AND NOTES

1. J. R. Clark, Chemistry of electronic gases. *J. Chem. Educ.* **83**, 857 (2006).

2. T. Alsop, Semiconductor industry sales worldwide 1987–2022 (2023); https://www.statista.com.

3. M. B. Chang, J. S. Chang, Abatement of PFCs from semiconductor manufacturing processes by nonthermal plasma technologies: A critical review. *Ind. Eng. Chem. Res.* **45**, 4101–4109 (2006).

4. H. M. Lee, S. H. Chen, Thermal abatement of perfluorocompounds with plasma torches. *Energy Procedia* **142**, 3637–3643 (2017).

5. W. T. Tsai, H. P. Chen, W. Y. Hsien, A review of uses, environmental hazards and recovery/recycle technologies of perfluorocarbons (PFCs) emissions from the semiconductor manufacturing processes. *J. Loss Prev. Process Ind.* **15**, 65–75 (2002).

6. A. J. Sicard, R. T. Baker, Fluorocarbon refrigerants and their syntheses: Past to present. *Chem. Rev.* **120**, 9164–9303 (2020).

7. R. T. Yang, *Gas Separation by Adsorption Processes* (World Scientific, 1997), vol. 1.

8. D. S. Sholl, R. P. Lively, Seven chemical separations to change the world. *Nature* **532**, 435–437 (2016).

9. M. B. Shiflett, D. R. Corbin, B. A. Elliott, S. Subramoney, K. Kaneko, A. Yokozeki, Sorption of trifluoromethane in activated carbon. *Adsorption* **20**, 565–575 (2014).

10. X. Huang, F. Chen, H. Sun, W. Xia, Z. Zhang, Q. Yang, Y. Yang, Q. Ren, Z. Bao, Separation of perfluorinated electron specialty gases on microporous carbon adsorbents with record selectivity. *Sep. Purif. Technol.* **292**, 121059 (2022).

11. D. Cao, S. Sircar, Heat of adsorption of pure sulfur hexafluoride on micro-mesoporous adsorbents. *Adsorption* **7**, 73–80 (2001).

12. J. Dunne, M. Rao, S. Sircar, R. Gorte, A. Myers, Calorimetric heats of adsorption and adsorption isotherms. 2. O_2, N_2, Ar, CO_2, CH_4, C_2H_6, and SF6 on NaX, H-ZSM-5, and Na-ZSM-5 zeolites. *Langmuir* **12**, 5896–5904 (1996).

13. I. M. Martos, J. Á. Ossorio, J. G. Sevillano, M. Doblaré, A. M. Calvo, S. Calero, Zeolites for the selective adsorption of sulfur hexafluoride. *Phys. Chem. Chem. Phys.* **17**, 18121–18130 (2015).

14. H. C. Zhou, J. R. Long, O. M. Yaghi, Introduction to metal-organic frameworks. *Chem. Rev.* **112**, 673–674 (2012).

15. H. Furukawa, K. E. Cordova, M. O’Keeffe, O. M. Yaghi, The chemistry and applications of metal-organic frameworks. *Science* **341**, 1230444 (2013).

16. S. Kitagawa, R. Kitaura, S.-I. Noro, Functional porous coordination polymers. *Angew. Chem. Int. Ed. Engl.* **43**, 2334–2375 (2004).

17. X. Zhao, Y. Wang, D. S. Li, X. Bu, P. Feng, Metal-organic frameworks for separation. *Adv. Mater.* **30**, 1705189 (2018).

18. J. R. Li, R. J. Kuppler, H. C. Zhou, Selective gas adsorption and separation in metal-organic frameworks. *Chem. Soc. Rev.* **38**, 1477–1504 (2009).

19. B. Moulton, M. J. Zaworotko, From molecules to crystal engineering: Supramolecular isomerism and polymorphism in network solids. *Chem. Rev.* **101**, 1629–1658 (2001).

20. D. K. Wanigarathna, J. Gao, B. Liu, Metal organic frameworks for adsorption-based separation of fluorocompounds: A review. *Mater. Adv.* **1**, 310–320 (2020).

21. M. E. Zick, J. H. Lee, M. I. Gonzalez, E. O. Velasquez, A. A. Uliana, J. Kim, J. R. Long, P. J. Milner, Fluoroarene separations in metal-organic frameworks with two proximal Mg^{2+} coordination sites. *J. Am. Chem. Soc.* **143**, 1948–1958 (2021).

22. P. J. Kim, Y. W. You, H. Park, J. S. Chang, Y. S. Bae, C. H. Lee, J. K. Suh, Separation of SF_6 from SF_6/N_2 mixture using metal-organic framework MIL-100(Fe) granule. *Chem. Eng. J.* **262**, 683–690 (2015).

23. J. Zheng, R. S. Vemuri, L. Estevez, P. K. Koech, T. Varga, D. M. Camaioni, T. A. Blake, B. P. McGrail, R. K. Motkuri, Pore-engineered metal-organic frameworks with excellent adsorption of water and fluorocarbon refrigerant for cooling applications. *J. Am. Chem. Soc.* **139**, 10601–10604 (2017).

24. M. B. Kim, S. J. Lee, C. Y. Lee, Y. S. Bae, High SF_6 selectivities and capacities in isostructural metal-organic frameworks with proper pore sizes and highly dense unsaturated metal sites. *Microporous Mesoporous Mater.* **190**, 356–361 (2014).

25. J. Zheng, D. Barpaga, B. A. Trump, M. Shetty, Y. Fan, P. Bhattacharya, J. J. Jenks, C. Y. Su, C. M. Brown, G. Maurin, B. P. McGrail, R. K. Motkuri, Molecular insight into fluorocarbon adsorption in pore expanded metal-organic framework analogs. *J. Am. Chem. Soc.* **142**, 3002–3012 (2020).

26. R. K. Motkuri, H. V. Annapureddy, M. Vijaykumar, H. T. Schaef, P. F. Martin, B. P. McGrail, L. X. Dang, R. Krishna, P. K. Thallapally, Fluorocarbon adsorption in hierarchical porous frameworks. *Nat. Commun.* **5**, 4368 (2014).

27. M. I. Hashim, H. T. Le, T. H. Chen, Y. S. Chen, O. Daugulis, C. W. Hsu, A. J. Jacobson, W. Kaveevivitchai, X. Liang, T. Makarenko, O. Š. Miljanić, I. Popovs, H. V. Tran, X. Wang, C. Wu, J. I. Wu, Dissecting porosity in molecular crystals: Influence of geometry, hydrogen bonding, and [π···π] stacking on the solid-state packing of fluorinated aromatics. *J. Am. Chem. Soc.* **140**, 6014–6026 (2018).

28. T. H. Chen, I. Popov, W. Kaveevivitchai, Y. C. Chuang, Y. S. Chen, O. Daugulis, A. J. Jacobson, O. Š. Miljanić, Thermally robust and porous noncovalent organic framework with high affinity for fluorocarbons and CFCs. *Nat. Commun.* **5**, 5131 (2014).

29. T. H. Chen, I. Popov, W. Kaveevivitchai, Y. C. Chuang, Y. S. Chen, A. J. Jacobson, O. Š. Miljanić, Mesoporous fluorinated metal-organic frameworks with exceptional adsorption of fluorocarbons and CFCs. *Angew. Chem. Int. Ed. Engl.* **54**, 13902–13906 (2015).

30. H. Wang, L. Yu, Y. Lin, J. Peng, S. J. Teat, L. J. Williams, J. Li, Adsorption of fluorocarbons and chlorocarbons by highly porous and robust fluorinated zirconium metal-organic frameworks. *Inorg. Chem.* **59**, 4167–4171 (2020).

31. J. Y. S. Lin, Molecular sieves for gas separation. *Science* **353**, 121–122 (2016).

32. Y. Wang, D. Zhao, Beyond equilibrium: Metal-organic frameworks for molecular sieving and kinetic gas separation. *Cryst. Growth Des.* **17**, 2291–2308 (2017).

33. T. L. Hu, H. Wang, B. Li, R. Krishna, H. Wu, W. Zhou, Y. Zhao, Y. Han, X. Wang, W. Zhu, Z. Yao, S. Xiang, B. Chen, Microporous metal-organic framework with dual functionalities for highly efficient removal of acetylene from ethylene/acetylene mixtures. *Nat. Commun.* **6**, 7328 (2015).

34. B. Li, X. Cui, D. O'Nolan, H. M. Wen, M. Jiang, R. Krishna, H. Wu, R. B. Lin, Y. S. Chen, D. Yuan, H. Xing, W. Zhou, Q. Ren, G. Qian, M. J. Zaworotko, B. Chen, An ideal molecular sieve for acetylene removal from ethylene with record selectivity and productivity. *Adv. Mater.* **29**, 1704210 (2017).

35. Z. Bao, J. Wang, Z. Zhang, H. Xing, Q. Yang, Y. Yang, H. Wu, R. Krishna, W. Zhou, B. Chen, Q. Ren, Molecular sieving of ethane from ethylene through the molecular cross-section size differentiation in gallate-based metal-organic frameworks. *Angew. Chem. Int. Ed. Engl.* **57**, 16020–16025 (2018).

36. R. B. Lin, L. Li, H. L. Zhou, H. Wu, C. He, S. Li, R. Krishna, J. Li, W. Zhou, B. Chen, Molecular sieving of ethylene from ethane using a rigid metal-organic framework. *Nat. Mater.* **17**, 1128–1133 (2018).

37. T. Ke, Q. Wang, J. Shen, J. Zhou, Z. Bao, Q. Yang, Q. Ren, Molecular sieving of C2-C3 Alkene from alkyne with tuned threshold pressure in robust layered metal-organic frameworks. *Angew. Chem. Int. Ed. Engl.* **59**, 12725–12730 (2020).

38. A. Cadiau, K. Adil, P. M. Bhatt, Y. Belmabkhout, M. Eddaoudi, A metal-organic framework-based splitter for separating propylene from propane. *Science* **353**, 137–140 (2016).

39. H. Wang, X. Dong, V. Colombo, Q. Wang, Y. Liu, W. Liu, X. L. Wang, X. Y. Huang, D. M. Proserpio, A. Sironi, Y. Han, J. Li, Tailor-made microporous metal-organic frameworks for the full separation of propane from propylene through selective size exclusion. *Adv. Mater.* **30**, e1805088 (2018).

40. L. Yu, X. Han, H. Wang, S. Ullah, Q. Xia, W. Li, J. Li, I. Da Silva, P. Manuel, S. Rudić, Y. Cheng, S. Yang, T. Thonhauser, J. Li, Pore distortion in a metal-organic framework for regulated separation of propane and propylene. *J. Am. Chem. Soc.* **143**, 19300–19305 (2021).

41. Y. Xie, Y. Shi, E. M. Cedeño Morales, A. El Karch, B. Wang, H. Arman, K. Tan, B. Chen, Optimal binding affinity for sieving separation of propylene from propane in an oxyfluoride anion-based metal-organic framework. *J. Am. Chem. Soc.* **145**, 2386–2394 (2023).

42. B. Liang, X. Zhang, Y. Xie, R. B. Lin, R. Krishna, H. Cui, Z. Li, Y. Shi, H. Wu, W. Zhou, B. Chen, An ultramicroporous metal-organic framework for high sieving separation of propylene from propane. *J. Am. Chem. Soc.* **142**, 17795–17801 (2020).

43. Q. Dong, Y. Huang, J. Wan, Z. Lu, Z. Wang, C. Gu, J. Duan, J. Bai, Confining water nanotubes in a $Cu_{10}O_{13}$-based metal-organic framework for propylene/propane separation with record-high selectivity. *J. Am. Chem. Soc.* **145**, 8043–8051 (2023).

44. S. Jiang, H. Sun, K. Gong, X. Huang, Y. Zhu, X. Feng, J. Xie, J. Liu, B. Wang, Metal-organic frameworks for breakthrough separation of 2-butene isomers with high dynamic selectivity and capacity. *Angew. Chem. Int. Ed. Engl.* **62**, e202302036 (2023).

45. A. H. Assen, Y. Belmabkhout, K. Adil, P. M. Bhatt, D. X. Xue, H. Jiang, M. Eddaoudi, Ultra-tuning of the rare-earth fcu-MOF aperture size for selective molecular exclusion of branched paraffins. *Angew. Chem. Int. Ed. Engl.* **54**, 14353–14358 (2015).

46. H. Wang, X. Dong, E. Velasco, D. H. Olson, Y. Han, J. Li, One-of-a-kind: A microporous metal-organic framework capable of adsorptive separation of linear, mono- and di-branched alkane isomers via temperature- and adsorbate-dependent molecular sieving. *Energy Environ. Sci.* **11**, 1226–1231 (2018).

47. D. D. Zhou, P. Chen, C. Wang, S. S. Wang, Y. Du, H. Yan, Z. M. Ye, C. T. He, R. K. Huang, Z. W. Mo, N. Y. Huang, J. P. Zhang, Intermediate-sized molecular sieving of styrene from larger and smaller analogues. *Nat. Mater.* **18**, 994–998 (2019).

48. L. Li, L. Guo, D. H. Olson, S. Xian, Z. Zhang, Q. Yang, K. Wu, Y. Yang, Z. Bao, Q. Ren, J. Li, Discrimination of xylene isomers in a stacked coordination polymer. *Science* **377**, 335–339 (2022).

49. D. O'Hagan, Understanding organofluorine chemistry. An introduction to the C-F bond. *Chem. Soc. Rev.* **37**, 308–319 (2008).

50. S. Mohanty, A. V. McCormick, Prospects for principles of size and shape selective separations using zeolites. *Chem. Eng. J.* **74**, 1–14 (1999).

51. X. W. Zhang, D. D. Zhou, J. P. Zhang, Tuning the gating energy barrier of metal-organic framework for molecular sieving. *Chem* **7**, 1006–1019 (2021).

52. D. Tanaka, K. Nakagawa, M. Higuchi, S. Horike, Y. Kubota, T. C. Kobayashi, M. Takata, S. Kitagawa, Kinetic gate-opening process in a flexible porous coordination polymer. *Angew. Chem. Int. Ed. Engl.* **47**, 3914–3918 (2008).

53. L. Bondorf, J. L. Fiorio, V. Bon, L. Zhang, M. Maliuta, S. Ehrling, I. Senkovska, J. D. Evans, J. O. Joswig, S. Kaskel, T. Heine, M. Hirscher, Isotope-selective pore opening in a flexible metal-organic framework. *Sci. Adv.* **8**, eabn7035 (2022).

54. C. Gu, N. Hosono, J. J. Zheng, Y. Sato, S. Kusaka, S. Sakaki, S. Kitagawa, Design and control of gas diffusion process in a nanoporous soft crystal. *Science* **363**, 387–391 (2019).

55. H. Zeng, M. Xie, T. Wang, R. J. Wei, X. J. Xie, Y. Zhao, W. Lu, D. Li, Orthogonal-array dynamic molecular sieving of propylene/propane mixtures. *Nature* **595**, 542–548 (2021).

56. Y. Chen, Y. Yang, Y. Wang, Q. Xiong, J. Yang, S. Xiang, L. Li, J. Li, Z. Zhang, B. Chen, Ultramicroporous hydrogen-bonded organic framework material with a thermoregulatory gating effect for record propylene separation. *J. Am. Chem. Soc.* **144**, 17033–17040 (2022).

57. Y. Yang, L. Li, R. B. Lin, Y. Ye, Z. Yao, L. Yang, F. Xiang, S. Chen, Z. Zhang, S. Xiang, B. Chen, Ethylene/ethane separation in a stable hydrogen-bonded organic framework through a gating mechanism. *Nat. Chem.* **13**, 933–939 (2021).

58. Q. Dong, X. Zhang, S. Liu, R. B. Lin, Y. Guo, Y. Ma, A. Yonezu, R. Krishna, G. Liu, J. Duan, R. Matsuda, W. Jin, B. Chen, Tuning gate-opening of a flexible metal-organic framework for ternary gas sieving separation. *Angew. Chem. Int. Ed. Engl.* **59**, 22756–22762 (2020).

59. X. Wang, R. Krishna, L. Li, B. Wang, T. He, Y. Z. Zhang, J. R. Li, J. Li, Guest-dependent pressure induced gate-opening effect enables effective separation of propene and propane in a flexible MOF. *Chem. Eng. J.* **346**, 489–496 (2018).

60. L. Li, R. B. Lin, R. Krishna, X. Wang, B. Li, H. Wu, J. Li, W. Zhou, B. Chen, Flexible-robust metal-organic framework for efficient removal of propyne from propylene. *J. Am. Chem. Soc.* **139**, 7733–7736 (2017).

61. J. L. Atwood, L. J. Barbour, A. Jerga, Storage of methane and freon by interstitial van der Waals confinement. *Science* **296**, 2367–2369 (2002).

62. S. M. Wang, X. T. Mu, H. R. Liu, S. T. Zheng, Q. Y. Yang, Pore-structure control in metal-organic frameworks (MOFs) for capture of the greenhouse gas SF_6 with record separation. *Angew. Chem. Int. Ed. Engl.* **61**, e202207066 (2022).

63. G. M. Sheldrick, SHELXT-Integrated space-group and crystal-structure determination. *Acta Crystallogr. A Found. Adv.* **71** (Pt 1), 3–8 (2015).

64. P. S. Bárcia, D. Guimarães, P. A. P. Mendes, J. A. C. Silva, V. Guillerm, H. Chevreau, C. Serre, A. E. Rodrigues, Reverse shape selectivity in the adsorption of hexane and xylene isomers in MOF UiO-66. *Microporous Mesoporous Mater.* **139**, 67–73 (2011).

65. F. Xu, Y. Yu, J. Yan, Q. Xia, H. Wang, J. Li, Z. Li, Ultrafast room temperature synthesis of GrO@HKUST-1 composites with high CO_2 adsorption capacity and CO_2/N_2 adsorption selectivity. *Chem. Eng. J.* **303**, 231–237 (2016).

66. H. R. Abid, Z. H. Rada, J. Shang, S. Wang, Synthesis, characterization, and CO_2 adsorption of three metal-organic frameworks (MOFs): MIL-53, MIL-96, and amino-MIL-53. *Polyhedron* **120**, 103–111 (2016).
